# Mechanical control of the alternative splicing factor PTBP1 regulates extracellular matrix stiffness induced proliferation and cell spreading

**DOI:** 10.1016/j.isci.2025.112273

**Published:** 2025-03-22

**Authors:** Pei-Li Tseng, Weiwei Sun, Ahmed Salem, Mubarak Alaklobie, Sarah C. Macfarlane, Annica K.B. Gad, Mark O. Collins, Kai S. Erdmann

**Affiliations:** 1 School of Biosciences, University of Sheffield, Sheffield S10 2TN, UK; 2 Department of Oncology and Metabolism, The Medical School, University of Sheffield, Sheffield S10 2TN, UK; 3 Department of Oncology-Pathology, Karolinska Institutet, Anna Steckséns gata 30A, 171 64 Solna, Sweden; 4 CQM - Centro de Química da Madeira, Universidade da Madeira, Campus da Penteada, 9020-105 Funchal, Portugal; 5 biOMICS Mass Spectrometry Facility, University of Sheffield, Sheffield S10 2TN, UK; 6 Department of Medical Laboratory Sciences, College of Applied Medical Sciences, University of Bisha, Bisha 67714, Saudi Arabia; 7 Department of Biomedical Laboratory Science, Sheba University, Sheba, Libya

**Keywords:** Cell biology, Organizational aspects of cell biology, Functional aspects of cell biology

## Abstract

Cells sense mechanical cues and convert them into biochemical responses to regulate biological processes such as embryonic development, aging, cellular homeostasis, and disease progression. In this study, we introduce a large-scale, systematic approach to identify proteins with mechanosensitive nuclear localization, highlighting their potential roles in mechanotransduction. Among the proteins identified, we focus here on the splicing factor PTBP1. We demonstrate that its nuclear abundance is regulated by mechanical cues such as cell density, size, and extracellular matrix (ECM) stiffness and that PTBP1 medicates the mechanosensitive alternative splicing of the endocytic adapter protein Numb. Furthermore, we show that PTBP1 and Numb alternative splicing is critical for ECM stiffness–induced epithelial cell spreading and proliferation as well as for mesenchymal stem cell differentiation into osteoblasts on a stiff matrix. Our results underscore the emerging role of alternative splicing in mechanotransduction and provide novel mechanistic insights into how matrix stiffness modulates cellular mechanoresponses.

## Highlights

• A screen to identify proteins with a mechanosensitive nuclear localization
• The nuclear abundance of the splicing factor PTBP1 is regulated by mechanical cues
• PTBP1 regulates mechanosensitive splicing of the endocytic adapter Numb
• PTBP1 is required for cellular mechanoresponses

## Graphical abstract

**Figure:**
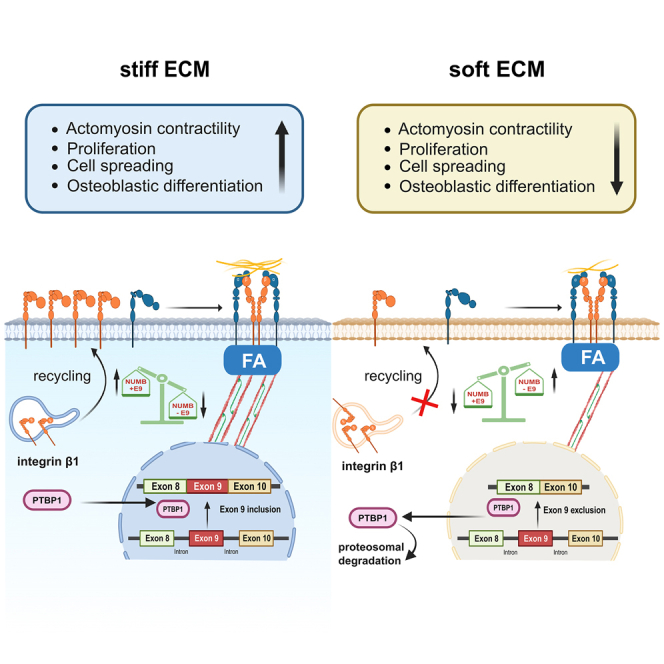

## Introduction

Mechanical properties of the cellular microenvironment (e.g., the stiffness of the extracellular matrix (ECM), fluid flow, and pressure) play important roles in cell differentiation, homeostasis, embryonic development, as well as in disease progression such as cancer or fibrosis.^1^^,^^2^^,^^3^ How mechanical cues are translated into biochemical cellular changes is still not fully understood. Plasma membrane resident mechanosensors such as integrins can sense extra- and intracellular forces and such mechanical forces are conducted through the cytoskeleton and the linker of nucleoskeleton and cytoskeleton complex (LINC) to the nucleus leading to changes in chromatin organization and nuclear pore complex permeability. This makes the nucleus a central player in mechanotransduction.^4^^,^^5^^,^^6^ Changes in chromatin structure can then have a direct effect on transcriptional activity whereas regulation of the permeability of the nuclear pore complex allows the shuttling of proteins between the nucleus and the cytoplasm, depending on their molecular size and mechanical stability.^4^^,^^7^^,^^8^ Mechanoresponsive nuclear transport receptors like importin-7 have further been identified to drive nuclear import of transcriptional regulators that play an important role in mechanotransduction.^9^ Thus, changes in the subcellular localization of proteins between the cytosol and the nucleus is a fundamental principle of mechanotransduction.^10^^,^^11^ Proteins that undergo mechanosensitive shuttling between the cytosol and the nucleus include transcriptional regulators such as the Yes 1 associated transcriptional regulator (YAP), the myocardin related transcription factor A (MRTF-A), the runt-related transcription factor 2 (Runx2), Snail, and Twist.^10^^,^^11^^,^^12^^,^^13^^,^^14^^,^^15^ Emerging evidence suggests that in addition to transcription factors, proteins that participate in other functions in the nucleus such as DNA-methylation can be regulated by mechanosensitive nuclear-cytoplasmic shuttling.^16^ Furthermore, the Src homology region 2-domain containing protein tyrosine phosphatase 1 (SHP1) has been shown to undergo nuclear shuttling by directly forming a complex with YAP, which is important for controlling tyrosine phosphorylation events in the nucleus.^17^ In this study, we have developed an unbiased screen to identify proteins, which demonstrate a mechanosensitive nuclear localization. Our approach integrates the controlled modulation of cellular actomyosin contractility with *in situ* proximity biotinylation of the nuclear proteome, followed by mass spectrometry analysis.^18^ Notably, the screen identified polypyrimidine tract binding protein 1 (PTBP1), a splicing regulator, as a novel mechanotransducer. We demonstrate that PTBP1 plays a crucial role in regulating mechanosensitive alternative splicing. Additionally, our findings provide new mechanistic insights into how ECM stiffness controls mesenchymal stem cell differentiation, cell spreading, and proliferation. Our results highlight the emerging importance of alternative mRNA splicing regulation as a key component of mechanotransduction.

## Results

### Design and validation of a screen to identify proteins with a mechanosensitive nuclear localization

**Figure 1:**
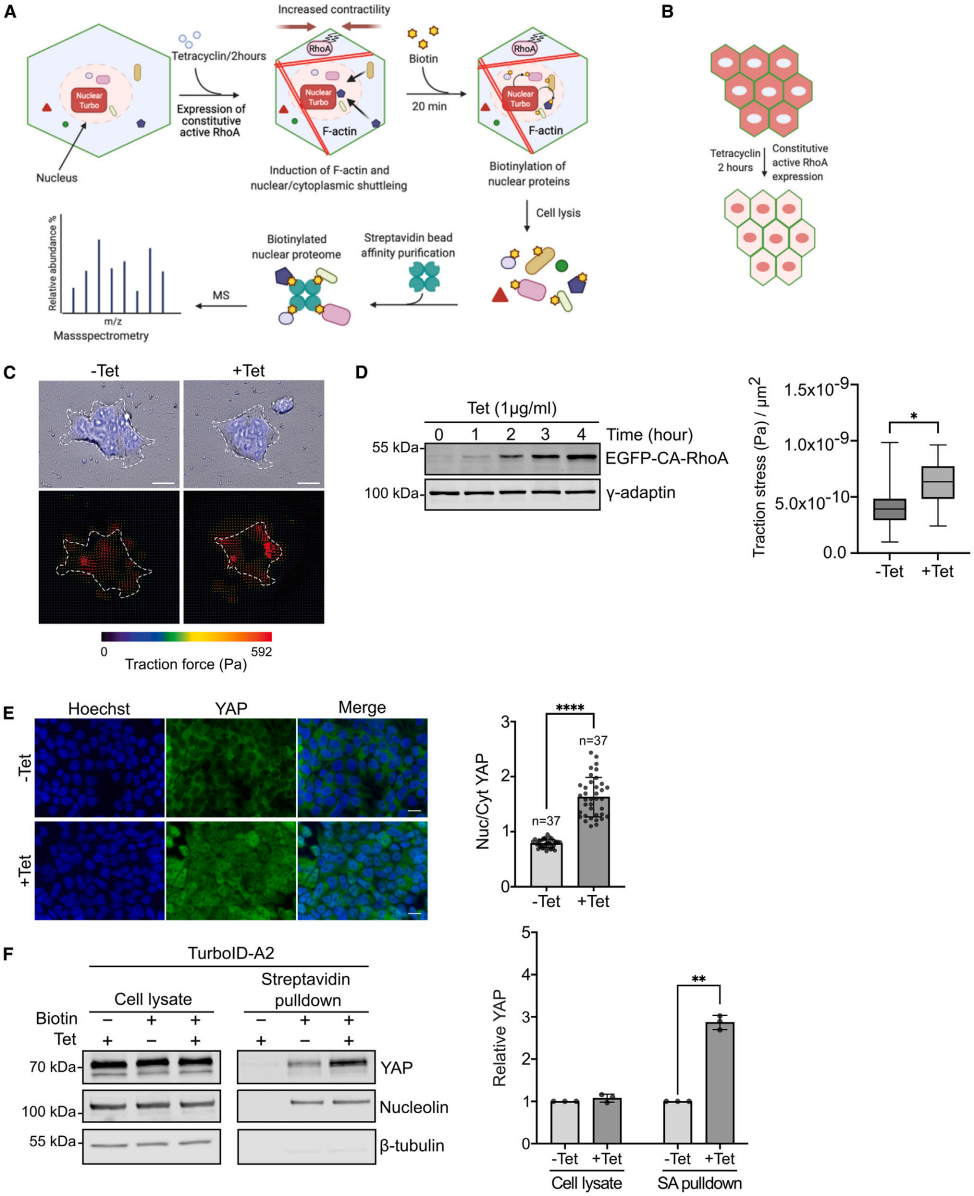
Screen approach to identify proteins potentially involved in mechanotransduction and its validation (A) Overview of the approach used for the screen. The diagram was created with BioRender.com. (B) Scheme of nuclear translocation induced by tetracycline induced contractility at high cell density. (C) Detection of traction force in HEK293-tet-RhoA before and after 2 h of tetracycline treatment. Cells were visualized by staining of nuclei. Red arrows indicated the force direction. Bar represents 20 μm. (D) Left panel: western blot showing HEK293-tet-RhoA expressed constitutively active RhoA by tetracycline induction in a time-dependent manner. γ-adaptin was used as loading control. Right panel: Quantification of traction stress in HEK293-tet-RhoA before and after tetracycline treatment. Data was analyzed by unpaired t-test. (E) Immunofluorescence and quantification of nuclear to cytoplasmic ratio of YAP in HEK293-tet-RhoA treated with or without tetracycline for 2 h. Bar represents 15 μm. Data were analyzed by unpaired t-test. (F) Biochemical purification of nuclear proteins using the approach described in (A). Biotinylated proteins were collected using streptavidin pulldown and probed by western blot using the indicated antibodies. Nucleolin and β-tubulin were used as nuclear and cytosolic marker respectively. Quantification shows normalized YAP expression. Data were analyzed by unpaired t-test. Replicates = 3 in all experiments. n = number of cells analyzed. Values are means ± s.d. ∗*p* < 0.05, ∗∗*p* < 0.01, ∗∗∗∗*p* < 0.0001.

To identify proteins potentially involved in mechanotransduction, we developed a screening approach that quantifies changes in the nuclear proteome following acute alterations in cellular actomyosin contractility, as outlined in Figures 1A and 1B. To enhance contractile forces in cells, we activated the small Rho GTPase RhoA, which leads to myosin light chain phosphorylation that increases the myosin motor activity and, thereby, elevating the cytoskeletal actomyosin-based contractile force of cells.^19^^,^^20^ The activation of RhoA has been previously employed to increase the traction and the intracellular tension of cells.^19^^,^^20^^,^^21^^,^^22^

For our screen, we first generated HEK293 cells which express a constitutively active variant of RhoA under a tetracycline-inducible promoter (HEK293-tet-RhoA). The expression of the constitutively active RhoA variant was observed as early as 1 h after tetracycline addition (Figure 1D), and a RhoA-induced increase in cellular traction was observed using traction force microscopy (Figures 1C and 1D). Next, we stably transfected a recently engineered biotin-ligase (NL-TurboID), which has an increased enzymatic activity and a nuclear localization signal,^18^ into HEK293-tet-RhoA cells (thereby, generating HEK293-tet-RhoA-TurboID). This approach allowed us to biotinylate selectively nuclear proteins *in situ* and to purify the biotinylated proteins via streptavidin-pulldown. Critically, this approach minimized potential alterations to the nuclear proteome caused by mechanical handling during standard subcellular fractionation methods. As expected, NL-TurboID localized to the nucleus (Figure S1A). We established efficient biotinylation of proteins via western blotting using two independent HEK293-tet-RhoA-TurboID clones (Figure S1B). To identify proteins enriched in the nucleus following the expression of the constitutively active RhoA, we compared the nuclear proteome of cells treated with tetracycline and those mock-treated for 2 h. The transcriptional regulator protein YAP served as a positive control. YAP is a well-known mechanotransducer that translocates to the nucleus in response to increased cellular contractile forces and extracellular stiffness.^10^^,^^11^ At high cell density, where actomyosin contractility is typically low, YAP was largely excluded from the nucleus. However, following RhoA activation, YAP translocated to the nucleus (Figure 1E). Using our biotinylation approach to purify nuclear proteins, we detected over a 3-fold enrichment of YAP in the nucleus of tetracycline-treated cells compared to mock-treated cells (Figures 1F and S1C). The purity of our fractions was confirmed by the presence of the nuclear marker nucleolin and the absence of the cytosolic marker tubulin. In summary, our data show that this screening approach successfully identifies RhoA-induced changes of protein levels in the nucleus, which provides a powerful tool for the discovery of novel mechanotransducers.

### The splicing regulator PTBP1 is a potential mechanotransducer

**Figure 2:**
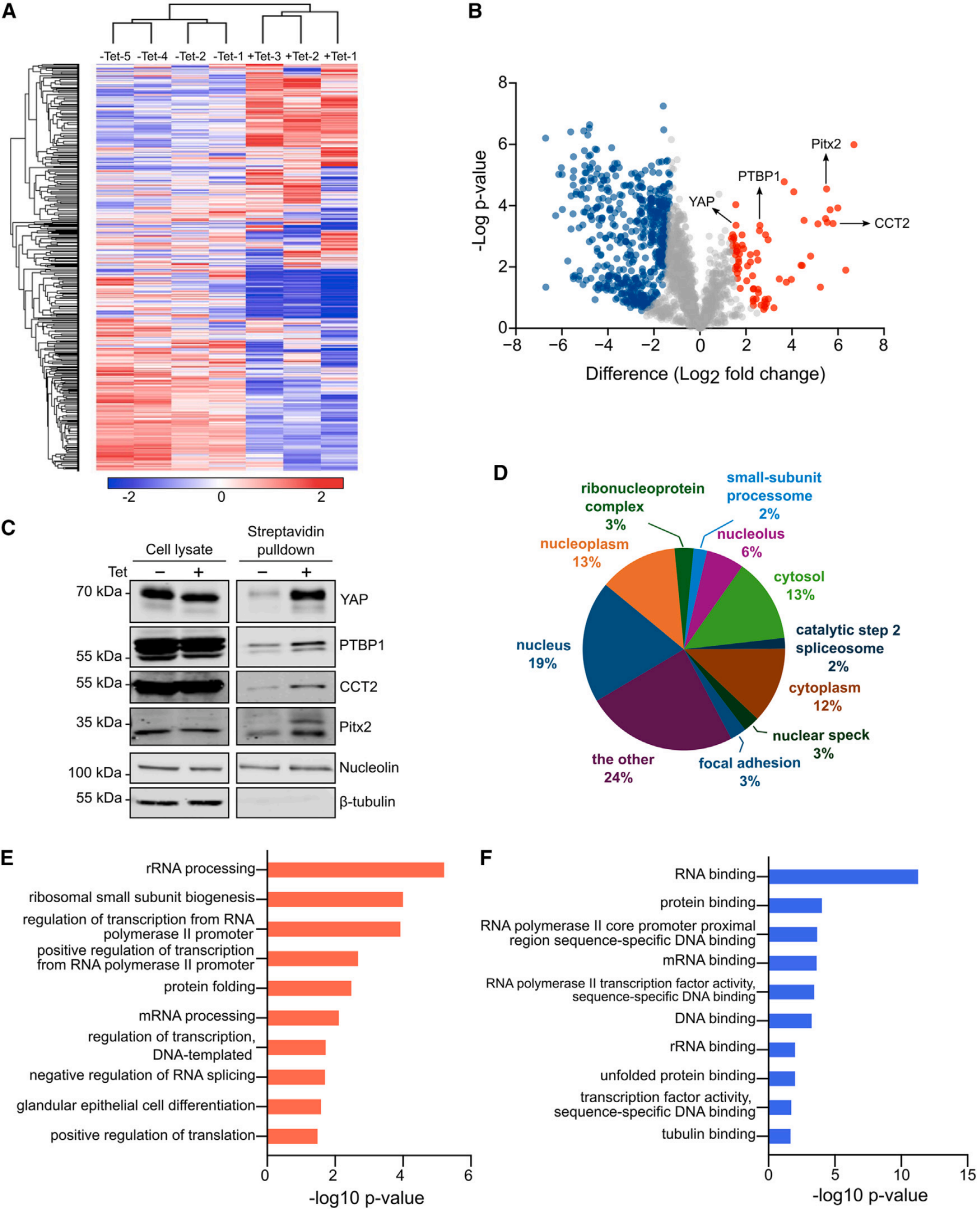
Nuclear proteome dynamics in response to constitutive active RhoA expression (A) Hierarchical cluster analysis of proteins showing increased or decreased abundance in the nucleus in HEK293-tet-RhoA-TurboID cells after 2 h tetracycline treatment. Rows represent clustering of proteins and columns represent the clustering of samples. (B) Volcano plot of proteins significantly enriched or reduced in the nucleus following the approach described in Figure 1A and using the same data as used for the hierarchical cluster analysis. Red dots in the right area represent proteins significantly increased in the nucleus after tetracycline treatment while blue dots indicate proteins significantly decreased in the nucleus by tetracycline treatment. (C) Validation of upregulated candidates identified in the volcano plot in B. Samples were prepared as described in Figure 1A and probed by western blot using indicated antibodies. (D) Distribution of main subcellular localizations of proteins found significantly increased in B. (E) Gene ontology based on biological process (GOBP) analysis of proteome data showing the top 10 GO terms with significant enrichment after tetracycline treatment. (F) Gene ontology based on molecular function (GOMF) analysis of proteome data revealed the top 10 most enriched GO terms after tetracycline treatment. All the proteome data were analyzed by DAVID database (https://david.ncifcrf.gov/) and the criteria of differentially enriched nuclear proteins as permutation-based FDR <0.05 with a Log2 nuclear enrichment >1.5.

To characterize changes in nuclear protein abundance following RhoA-activation, we used label-free quantitative mass spectrometry to measure the relative abundance of streptavidin-purified proteins (Table S1). Hierarchical cluster analysis revealed that RhoA-activation significantly altered the profiles of nuclear proteins (Figure 2A). We identified 74 proteins that were enriched in the nucleus by RhoA-activation. The observation that these included YAP, which was identified with high confidence, validates our overall approach (Figure 2B). Using the same approach, we also identified 543 proteins that were less abundant in the nucleus upon RhoA-activation. To further validate the mass spectrometry results, we quantified the nuclear enrichment of selected proteins through nuclear fractionation, followed by western blot analysis. This analysis showed that RhoA enriched the splicing regulator PTBP1, molecular chaperone CCT2, and transcription factor Pitx2 in the nucleus. As expected, YAP, the positive control, also displayed increased nuclear localization upon RhoA activation, further validating our approach (Figure 2C).

Although many of the nuclear-enriched proteins were already known to be primarily nuclear, we also identified several proteins known to localize to both the cytoplasm and nucleus (Figure 2D). This supports the hypothesis that proteins shuttle between the nucleus and cytoplasm and vice versa in response to tetracycline-induced actomyosin contractility. Gene ontology analysis of the upregulated nuclear proteins revealed enrichment in processes such as RNA processing, RNA metabolism, and transcriptional regulation, including mRNA splicing (Figures 2E and 2F). This aligns with growing evidence that mechanical cues can regulate alternative mRNA splicing through so far poorly understood mechanisms.^23^^,^^24^ Given that PTBP1 has previously been shown to shuttle between the nucleus and cytoplasm and its established role in alternative splicing regulation, we decided to further investigate its potential role in mechanotransduction.^25^

### Nuclear localization of PTBP1 is regulated via cell density, cell size, ECM stiffness, and actomyosin contractility

Our screen for identifying novel mechanotransducers is based on the activation of RhoA, which in addition to regulating actomyosin contractility also controls other cellular functions and signaling pathways.^26^ To determine whether the observed effects on protein localization to the nucleus were regulated by mechanical cues, we hypothesized that proteins involved in mechanotransduction would behave similarly to the established mechanotransducer, YAP. In cells cultured at high density, on small-area micropatterns, or on soft ECM, YAP is predominantly excluded from the nucleus, whereas it becomes enriched in the nucleus at low cell density, on large-area micropatterns, or on stiff ECM.^10^^,^^27^

**Figure 3:**
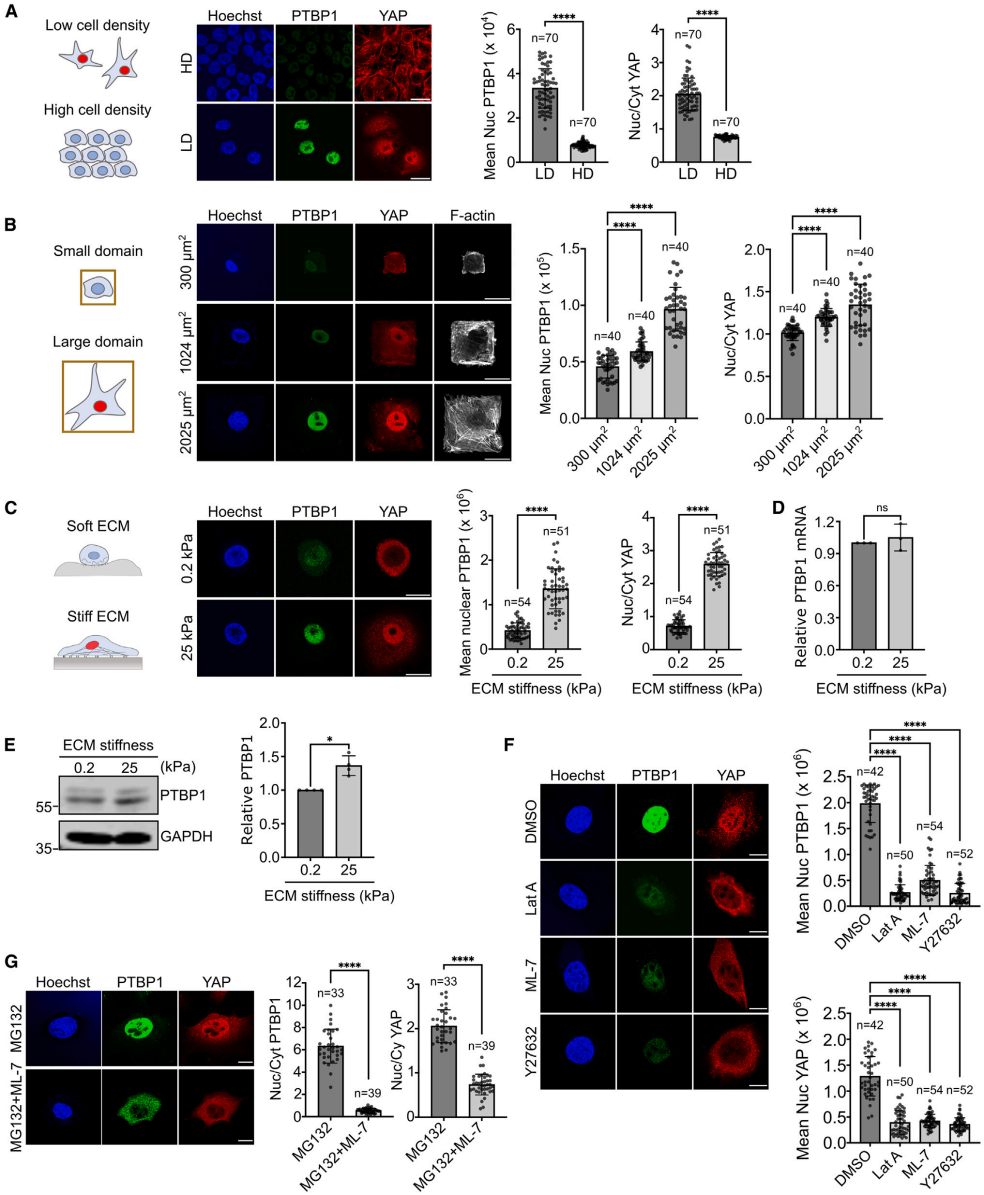
Nuclear abundance of PTBP1 is regulated by mechanical cues (A) Left and Middle: MCF10A cells were plated at high and low cell density and subcellular localization of PTBP1 and YAP was determined by immunofluorescence using the indicated antibodies. Right: Quantification shows the nuclear intensity for PTBP1 and the nuclear to cytoplasmic ratio for YAP at low cell density (LD) or high cell density (HD). Data were analyzed by unpaired t-test. (B) Mesenchymal stem cells (MSC) were plated on fibronectin coated micropattern of defined size. PTBP1 and YAP in single cell of each domain were measured using immunofluorescence microscopy. Quantification was performed by ordinary one-way ANOVA. (C) Left and Middle: 1x10^5^ MCF10A were plated on 35mm of soft (0.2kPa) or stiff (25kPa) collagen coated PAA gels to reach low cell density and the subcellular localization of PTBP1 and YAP was quantified as described in (A). (D) Quantitative PCR for PTBP1 mRNA from MCF10A cultured either on soft (0.2kPa) or stiff (25kPa) matrix. Data were analyzed by unpaired t-test. (E) Western blot for PTBP1 using total protein extracts from MCF10A cells cultured on soft or stiff matrix. Data analysis was performed by unpaired t-test. Experiment replicates = 4. (F) NIH3T3 fibroblasts were cultured at low cell density on plastic (stiff) and either treated with DMSO (control), Latrunculin A (Lat.A), Myosin light-chain kinase inhibitor-7 (ML-7) or the Rho kinase inhibitor Y27632 for 30 min. Cells were fixed and indicated proteins were identified by immunofluorescence. The mean nuclear intensities for PTBP1 and YAP after incubation with the inhibitors was quantified. Data were analyzed by ordinary one-way ANOVA. (G) NIH3T3 fibroblasts were incubated with the indicated inhibitors or combination of inhibitors and the subcellular localization of PTBP1 and YAP were assessed using immunofluorescence. The nuclear/cytosol ratio for PTBP1 and YAP in cells treated with indicated inhibitor was quantified. Data were analyzed by unpaired t-test. Replicates = 3 in all experiments unless stated otherwise. n = numbers of cells analyzed. Values are means ± s.d. ∗*p* < 0.05, ∗∗∗∗*p* < 0.0001, ns: not significant. Bar in immunofluorescence images of A-C represents 20 μm, in images of F-G represents 10 μm.

Using non-transformed MCF10A breast epithelial cells and immunofluorescence analysis, we observed that the abundance of PTBP1 in the nucleus decreases at high cell density but increases at low cell density, similar to the behavior of YAP (Figure 3A). However, unlike YAP, there was no significant increase in PTBP1 levels in the cytosol at high cell density. The specificity of the antibodies against PTBP1 and YAP was validated by siRNA (Table S2) which specifically targeted these proteins (Figures S2A–S2C).

Next, we cultured mesenchymal stem cells on fibronectin-coated micropatterns of varying sizes and found that the nuclear fluorescence intensity of PTBP1 increased with the size of the pattern. This observation mirrored the results obtained at different cell densities, with only little increase in cytosolic PTBP1 on the smallest micropattern (Figure 3B). Additionally, we examined PTBP1 localization on soft and stiff ECM (collagen-coated polyacrylamide (PAA) gels with varying stiffness) and found higher nuclear PTBP1 levels on stiff ECM compared to soft ECM (Figure 3C). We confirmed this stiffness-dependent nuclear localization of PTBP1 in primary human mammary epithelial cells, human mesenchymal stem cells, as well as in HeLa cells (Figures S2D–S2F).

To assess whether ECM stiffness regulates PTBP1 at the mRNA level, we performed quantitative PCR on RNA isolated from cells cultured on soft or stiff ECM and found no difference in PTBP1 mRNA levels (Figure 3D, Table S3). A comparison of PTBP1 protein levels revealed a significant reduction in PTBP1 in cells cultured on soft ECM, suggesting that PTBP1 regulation occurs at the protein level rather than at the transcriptional level (Figure 3E).

Since cell density, cell area, and ECM stiffness influence proliferation rates, we analyzed whether the changes in nuclear PTBP1 reflected the proliferation state of the cells, rather than their mechanical environment. When we blocked proliferation with doxorubicin and analyzed nuclear PTBP1, we found no changes in the levels of nuclear PTBP1. The successful block of proliferation was confirmed by the proliferation marker Ki67 (Figures S2G and S2H).

Given the similar behaviors of PTBP1 and YAP, we investigated whether there is interdependence between these proteins. To do so, we assessed whether PTBP1 knockdown on a stiff matrix affects the localization of YAP or its closely related family member, TAZ. Interestingly, PTBP1 knockdown led to a reduction in nuclear YAP levels, accompanied by an increase in its cytosolic localization. In contrast, no significant changes were observed in TAZ localization. Furthermore, in reciprocal experiments involving YAP or TAZ knockdown, nuclear PTBP1 levels remained unchanged (Figure S2J). These findings suggest that PTBP1 may function upstream of YAP.

We then investigated more directly whether PTBP1 subcellular localization is regulated by actomyosin contractility, as previously described for YAP.^10^ NIH3T3 fibroblasts were cultured on stiff plastic surfaces and treated with inhibitors of actomyosin contractile forces, including the actin polymerization inhibitor latrunculin A, the myosin light-chain kinase inhibitor ML-7, and the Rho kinase inhibitor Y27632. As expected, these inhibitors reduced YAP nuclear localization and increased its cytoplasmic levels. Similarly, we observed a significant reduction in nuclear PTBP1. However, unlike YAP, we did not observe cytosolic enrichment of PTBP1 (Figure 3F).

We hypothesized that PTBP1 might undergo rapid degradation in the cytosol, given the short experimental time frame. To test this, we blocked protein degradation via the proteasome in the presence of ML-7. Inhibition of protein degradation alone did not affect PTBP1 or YAP localization. However, when protein degradation via the proteasome was inhibited in conjunction with ML-7 treatment, PTBP1 levels significantly decreased in the nucleus and increased in the cytosol. This suggests that PTBP1 is rapidly degraded after being translocated from the nucleus to the cytosol (Figure 3G). In summary, our data suggest that the nuclear abundance of PTBP1 is regulated by mechanical cues such as cell density, cell size, and ECM stiffness via actomyosin contractility. Therefore, PTBP1 is a potential key player in cellular mechanotransduction.

### PTBP1 mediates cellular mechanoresponses

**Figure 4:**
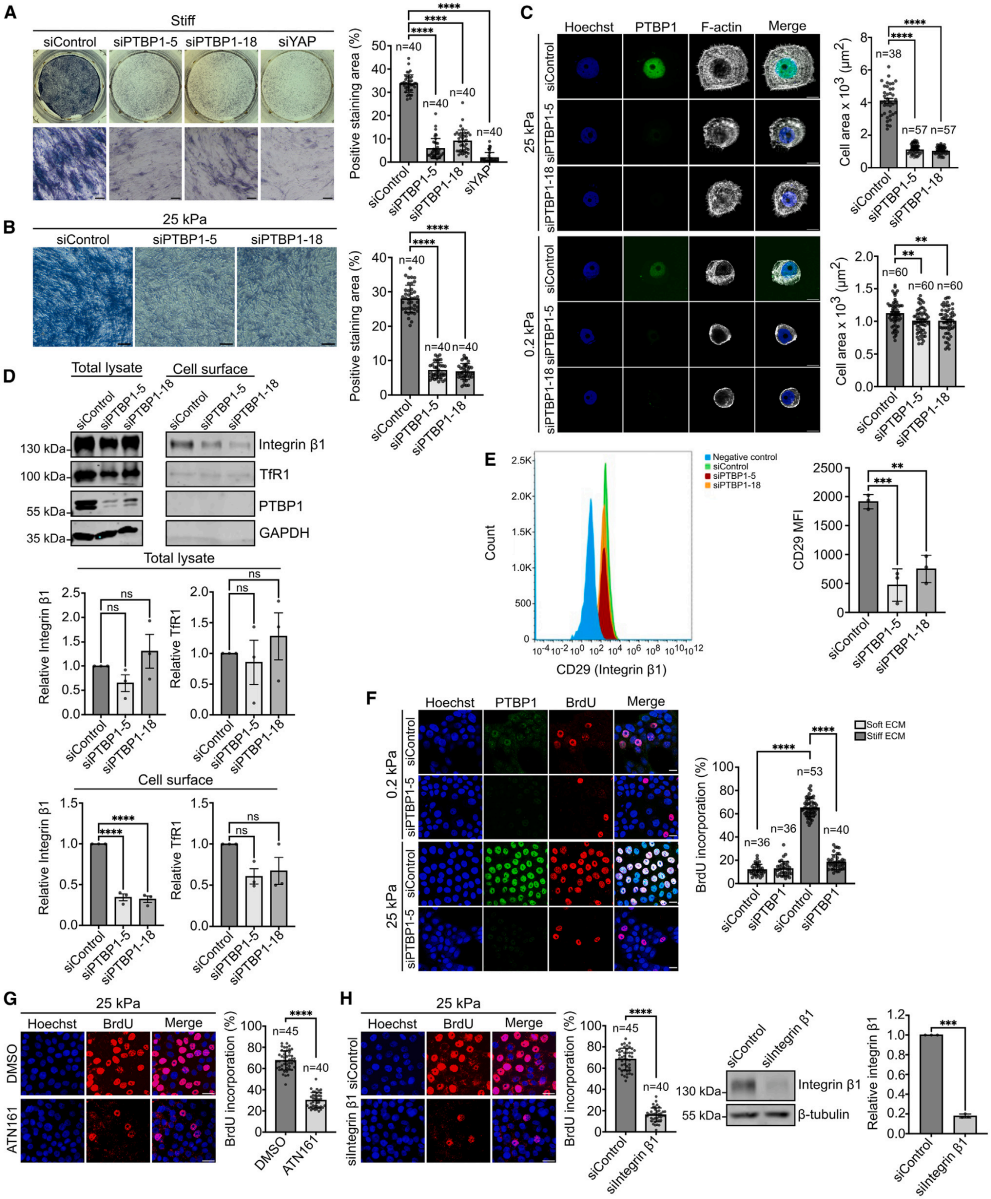
PTBP1 is important for cellular mechanoresponses (A) Mesenchymal stem cells were cultured on plastic (stiff) and transfected with the indicated siRNAs. Osteoblastic differentiation was quantified using alkaline phosphatase staining. Bar represents 100 μm and refers to the bottom images. Data were analyzed using ordinary one-way ANOVA. (B) Mesenchymal stem cells were cultured on collagen coated stiff PAA gels (25 kPa), transfected with the indicated siRNAs and osteoblastic differentiation was quantified as described in (A). Bar represents 100 μm. (C) MCF10A cells transfected with the indicated siRNAs and seeded on stiff (25 kPa) or soft (0.2 kPa) collagen coated PAA gels. Cells were stained with F-actin for measurement of spreading area. Knockdown was confirmed by PTBP1 immunofluorescence. Bar represents 10 μm. Data were analyzed using ordinary one-way ANOVA. (D) MCF10A cells were transfected with the indicated siRNAs followed by a surface biotinylation assay. Total proteins and cell surface proteins were analyzed by western blot using indicated antibodies. Bar diagrams represent the quantification of the experiments. Data were analyzed by ordinary one-way ANOVA. (E) MCF10A cells were transfected with the indicated siRNAs and the surface amount of integrin β1 (CD29) was quantified using flow cytometry. Data were analyzed by ordinary one-way ANOVA. (F) MCF10A cells were cultured on stiff (25 kPa) or soft (0.2 kPa) collagen coated PAA gels and transfected with the indicated siRNAs. Proliferation rate and knockdown was determined by immunofluorescence. Bar diagram shows the quantification of BrdU incorporation. Bar represents 20 μm. Data were analyzed by ordinary one-way ANOVA. (G) MCF10A cells were cultured on stiff (25 kPa) collagen coated PAA gels and treated with 50 μM ATN161 inhibitor overnight. Proliferation rate was quantified using the BrdU assay. Bar represents 50 μm. Data were analyzed by unpaired t-test. (H) MCF10A cells were seeded on stiff (25 kPa) collagen coated PAA gels and transfected with the indicated siRNAs. Knockdown was confirmed by western blot and proliferation rate was quantified by BrdU assay. Bar represents 50 μm. Data were analyzed by unpaired t-test. Three replicates in all experiments. n = numbers of cells analyzed or in the case of BrdU assays refers to the number of analyzed microscopic fields. All values are means ± s.d. ∗∗*p* < 0.01, ∗∗∗*p* < 0.001, ∗∗∗∗*p* < 0.0001, ns: not significant.

To directly assess a possible role of PTBP1 in cellular mechanoresponse, we first investigated the role of PTBP1 in mesenchymal stem cell differentiation, a process which is regulated by ECM stiffness.^28^ Mesenchymal stem cells are known to differentiate into osteoblasts on stiff matrices, whereas softer matrices promote adipocyte differentiation. This process is controlled by the stiffness-dependent nuclear localization of the transcriptional regulator YAP.^10^ Our observation that also PTBP1 is accumulated in the nucleus on stiff matrices but not on soft matrices in mesenchymal stem cells prompted us to investigate PTBP1’s role in mesenchymal stem cell differentiation (Figure S2D). To test this, we cultured mesenchymal stem cells on a stiff matrix (plastic) followed by siRNA-mediated knockdowns of PTBP1 and YAP, using YAP as a positive control. Osteoblast differentiation was evaluated using an alkaline phosphatase assay. The results showed a significant reduction in osteoblastic differentiation following PTBP1 knockdown, similar to the reduction observed after YAP knockdown (Figure 4A). PTBP1 knockdown in mesenchymal stem cells was controlled by western-blotting (Figure S2I). We also observed comparable effects on osteoblast differentiation, when PTBP1 was knocked down in cells grown on stiff collagen-coated PAA gels (Figure 4B). These findings suggest that PTBP1 facilitates osteoblast differentiation on a stiff matrix.

Next, we explored PTBP1’s role in another cellular process influenced by ECM stiffness: cell spreading. Typically, a stiff matrix enhances the spreading of cells. To test a possible role of PTBP1 in cell spreading, MCF10A cells were transfected with PTBP1-targeting siRNAs, and the effect on cell spreading was evaluated on both stiff and soft collagen-coated PAA gels. We observed a significant reduction in cell spreading on a stiff matrix and to a much lesser extend also on soft matrix after PTBP1 knockdown. The knockdown was confirmed by immunofluorescence (Figure 4C).

The observed reduced cell spreading on collagen-coated matrices prompted us to investigate the total and cell surface expression levels of integrin β1, a key molecule in cell spreading on collagen I.^29^^,^^30^ Using cell surface biotinylation assays and flow cytometry, we observed that PTBP1 knockdown reduced the protein levels of integrin β1 at the cell surface, although the total levels of the protein remained largely unchanged (Figure 4D and 4E).

Cell spreading and integrin β1 expression have been closely linked to cell proliferation, another process known to be influenced by ECM stiffness.^31^^,^^32^^,^^33^ To determine if PTBP1 plays a role in stiffness-induced cell proliferation, we analyzed the proliferation rates of MCF10A cells in which PTBP1 was knocked down, and control transfected cells, cultured on soft or stiff collagen-coated polyacrylamide gels. As expected, for all cells, the proliferation was significantly higher on the stiff matrix compared to the soft matrix. Importantly, while PTBP1 knockdown had no effect on proliferation on soft ECM, it significantly reduced proliferation on stiff ECM, nearly to the level observed on soft matrices (Figure 4F). This observation suggests that PTBP1 is required for stiffness-induced cell proliferation. Finally, we asked the question whether blocking integrin β1 binding to collagen, or downregulation of integrin β1 is sufficient to inhibit proliferation on a stiff matrix. To test this, we disrupted the binding of integrin β1 to collagen using the small molecule inhibitor ATN161 or reduced integrin β1 expression level using siRNA (Table S2), respectively. Both approaches led to a significant reduction in the proliferation on stiff matrix (Figures 4G and 4H). In summary, our data suggest that PTBP1 is important for several cellular mechanoresponses, which establishes PTBP1 as a novel mechanotransducer.

### PTBP1 controls the mechanosensitive splicing of the endocytic adapter protein Numb

**Figure 5:**
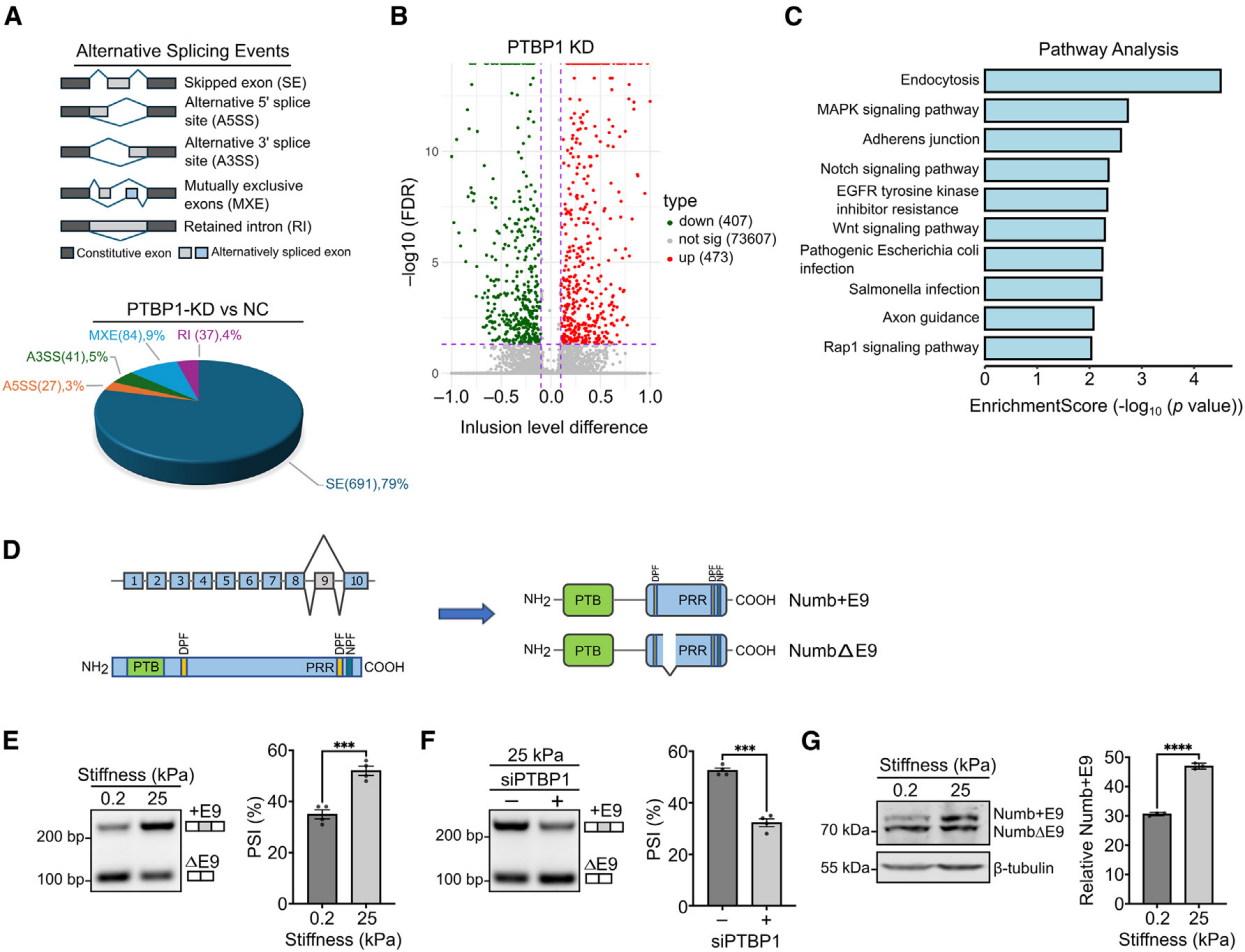
PTBP1 regulates matrix stiffness dependent splicing of Numb (A) Percentage and absolute number of changed alternative splicing events after PTBP1 knockdown categorized by splicing type. (B) Volcano plot showing up or downregulated alternative splicing events after PTBP1 knockdown. (C) Pathway analysis of all changed alternative splicing events after PTBP1 knockdown. (D) Top left: Exon-intron structure of Numb. Bottom left: Modular structure of the Numb protein, PTB = phosphotyrosine binding domain, PRR = proline rich region. Right: Modular structure of Numb showing the localization of the 49 amino acid inserts encoded by exon 9. (E) MCF10A cells were cultured on stiff (25 kPa), or soft (0.2 kPa) collagen coated PAA gels for 5 days. Expression of exon 9 inclusion (+E9) or exclusion (ΔE9) mRNA isoform was assessed using reverse transcription followed by PCR. The percent splicing inclusion (PSI) of +E9 isoform was calculated as the inclusion level (%) of the +E9 isoform over the sum of +E9 and ΔE9 isoforms. Experiment replicates = 4. Statistical analysis was performed by unpaired t-test. (F) MCF10A cells were cultured on stiff (25 kPa) collagen coated PAA gels and transfected with the siRNA targeting PTBP1 or control siRNA. The PSI (%) of exon 9 mRNA was determined as described in (E). Statistical analysis was performed by unpaired t-test. Experiment replicates = 4. (G) MCF10A cells cultured on stiff (25 kPa) or soft (0.2 kPa) collagen coated PAA gels for 5 days. Total protein was isolated and analyzed by western blot using the indicated antibodies. β-tubulin was used as loading control. Exon 9 inclusion was determined by densitometric quantification of corresponding western blot. Replicates = 3 in all experiments unless stated otherwise. Data were analyzed by unpaired t-test. All values are means ± s.d. ∗∗∗*p* < 0.001, ∗∗∗∗*p* < 0.0001.

PTBP1 is a main regulator of mRNA alternative splicing.^34^ To address the mechanism how PTBP1 contributes to mechanoresponse, we first identified PTBP1 alternative splicing targets in MCF10A cells. For this we performed PTBP1 knockdown experiments followed by RNA sequencing analysis. This analysis revealed 880 changes in alternative splicing across all alternative splicing types, including skipped exons, mutually exclusive spliced exons, alternative 5′ splice sites, alternative 3′ splice sites, and intron retention, with skipped exons the most abundant type of splicing events (Figure 5A). PTBP1 promotes exon inclusion as well as exclusion depending on the specific splicing events (Figure 5B). The quality of the RNA sequencing data was validated through semi-quantitative PCR of several alternative splicing events, which overall showed a strong correlation with the RNA sequencing results (R^2^ = 0.94) (Figure S3A and S3B). Pathway analysis of PTBP1-regulated alternative splicing events indicated an enrichment of alternative splicing of genes involved in endocytosis (Figure 5C). Notably, among the genes undergoing PTBP1-regulated alternative splicing in MCF10A cells, we identified the endocytic adapter Numb, previously shown to regulate cell spreading, integrin cell surface expression, and proliferation in an isoform-specific manner (Figure S3A).^35^ Thus, we chose Numb as a promising candidate for further investigation.

Numb is a multifunctional adapter protein involved in the regulation of cell fate decisions, endocytosis, and proliferation.^36^ It consists of an N-terminal phosphotyrosine binding (PTB) domain and a C-terminal proline-rich region (PRR). The Numb gene is composed of 10 exons, with PTBP1 regulating the alternative splicing of exon 9, leading to the addition of 46 amino acids to the proline-rich region of the protein (Figure 5D). To determine whether the PTBP1-dependent alternative splicing of exon 9 in Numb is influenced by mechanical cues, we cultured MCF10A breast epithelial cells on stiff or soft collagen-coated PAA gels and quantified exon 9 inclusion in the mRNA via polymerase chain reaction. We observed increased exon 9 inclusion in cells grown on stiff ECM compared to those grown on soft ECM (Figure 5E), which is consistent with our observation that a stiff underlying matrix increases the levels of PTBP1 in the nucleus. As predicted by our RNA sequencing results, this increase in alternative splicing on a stiff matrix was inhibited by PTBP1- targeting siRNA (Figure 5F), which indicates that PTBP1 is required for ECM stiffness-dependent alternative splicing of the Numb mRNA. Notably, also other PTBP1 targets identified in our RNA sequencing approach, such as the myosin MYO9A or the adapter protein ABI1, showed similar matrix stiffness-dependent alternative splicing (Figure S3C, Table S4), suggesting that PTBP1 is regulating a set of stiffness-dependent alternative splicing events. However, not all PTBP1 targets display stiffness-dependent regulation, which points to a more complex mechanism, which likely involves the regulation of additional splicing factors that compete for targets.

Next, we investigated whether the splicing changes observed at the mRNA level for Numb are shown at the protein level. For this, MCF10A cells were cultured on soft and stiff ECM, followed by analysis of the expression of Numb isoform using western blotting. We detected two variants of Numb, appearing as a doublet band, where the upper band corresponds to the protein isoform(s) including the additional 46 amino acids encoded by exon 9. Importantly, stiff culture conditions increased the expression of the longer splicing isoform of Numb (Figure 5G). These findings suggest that ECM stiffness can regulate the ratio between the isoforms of the Numb protein, and that it favors the longer splicing variant over the shorter one.

### Alternative splicing of Numb regulates cellular mechanoresponse

**Figure 6:**
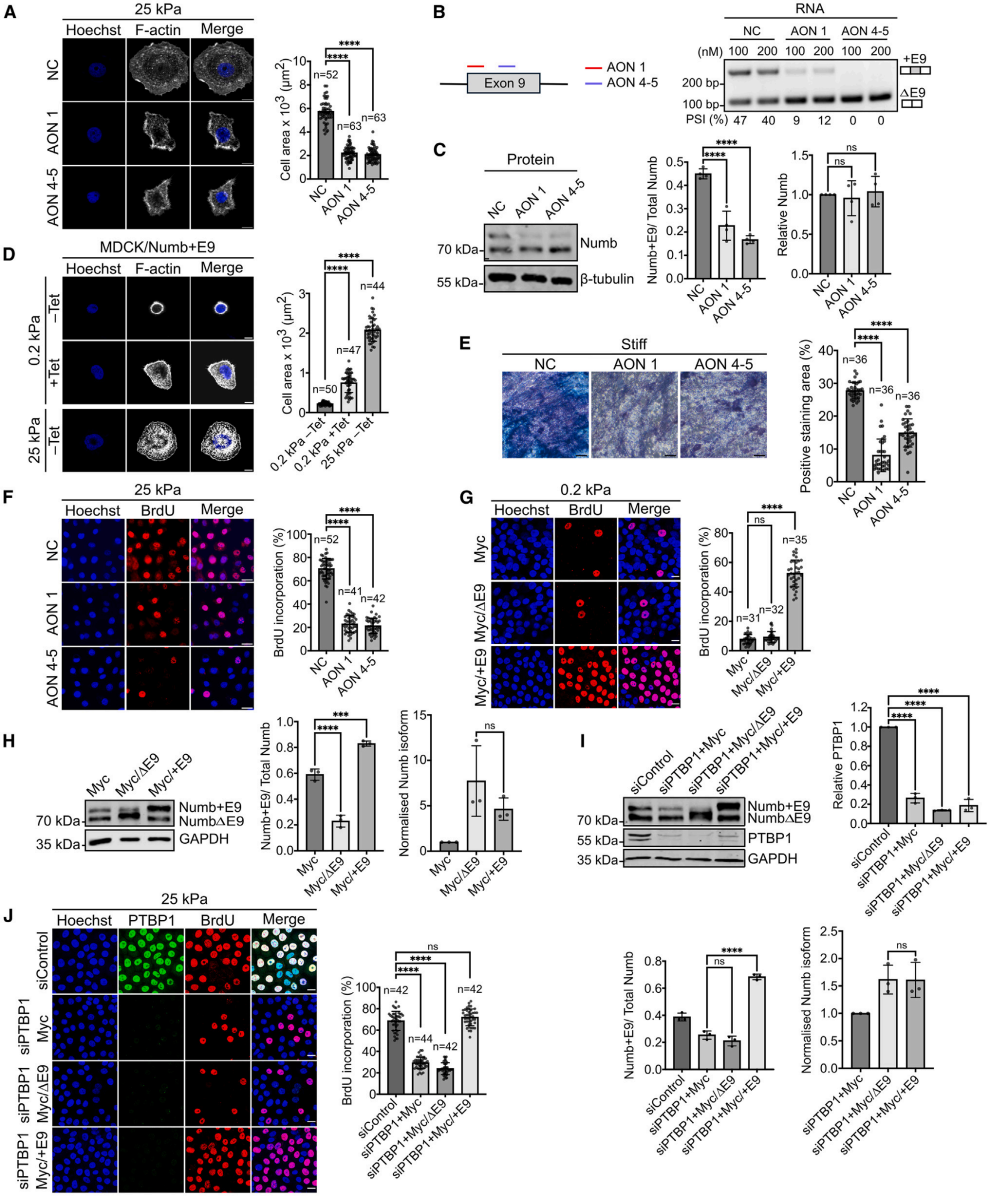
Alternative splicing of Numb controls cellular mechanoresponses (A) MCF10A cells were transfected with the indicated antisense oligonucleotides (AONs) and seeded on stiff (25 kPa) collagen coated PAA gels. Cells were stained for F-actin and spread area was quantified. Bar represents 10 μm. (B) MCF10A cells were transfected with the indicated AONs and alternative splicing changes were assessed by RT-PCR. (C) Total protein lysate from MCF10A transfected with indicated AONs was analyzed by western blot using the indicated antibodies. Bar plot shows the quantification of relative Numb+E9 isoform and total Numb protein expression, respectively. Experiments = 4. (D) MDCK cells stably expressing the +E9 Numb isoform under a tetracycline inducible promoter were treated with (+) or without tetracycline (−) for 4 h and plated on soft (0.2 kPa) or stiff (25 kPa) collagen coated PAA gels for 20 h. Cells were fixed and stained with F-actin and the spread area was measured. Bar represents 10 μm. (E) Mesenchymal stem cells were cultured on stiff matrix (plastic) and transfected with the indicated AONs or AON scramble (NC). Osteoblastic differentiation was quantified by an alkaline phosphatase assay. Bar represents 100 μm. (F) MCF10A cells were cultured on stiff (25 kPa) PAA gels and transfected with the indicated AONs. Cell proliferation was quantified using a BrdU assay. Bar represents 50 μm. (G) MCF10A cells were transfected with the isoform specific Numb expression constructs and plated on soft (0.2 kPa) collagen coated PAA gels. Cell proliferation was measured using a BrdU assay. Bar represents 20 μm. (H) Quantification of overexpression of Numb isoforms of the experiment described in (G) via western blotting. Left bar diagram shows quantification of Numb+E9 isoform overexpression as percentage of Numb+E9 of total Numb expression. Right bar diagram shows the relative expression of each Numb isoforms normalized to the level of NumbΔE9 of Myc control. (I) MCF10A were cultured on 25 kPa PAA gels and transfected with indicated siRNA and Numb isoform specific expression constructs. Upper panel: Western blot confirms PTBP1 knockdown. Lower panel: Quantification of Numb overexpression as described in (H). (J) Cell proliferation of cells analyzed in I was quantified using BrdU assay. Bar represents 20 μm. Replicates = 3 in all experiments unless stated otherwise. n = numbers of cells analyzed or in the case of BrdU assays refers to the number of analyzed microscopic fields. All data analysis was performed by ordinary one-way ANOVA. Values are means ± s.d. ∗∗∗*p* < 0.001, ∗∗∗∗*p* < 0.0001, ns: not significant.

To investigate the potential role of alternative splicing in mediating cellular mechanoresponse, we altered the ratio of Numb isoforms, by employing two independent antisense oligonucleotides (Table S2), which prevent the inclusion of exon 9 without changing total Numb expression levels (Figures 6B and 6C). Transfection of MCF10A cells with these antisense oligonucleotides phenocopied the knockdown of PTBP1 and significantly reduced cell spreading on stiff matrix (Figure 6A). Similar results were obtained by using isoform-specific siRNAs against Numb, which resulted in a significant decrease in cell spreading when the longer isoform was specifically targeted, whereas targeting the shorter isoform had no impact on cell spreading (Figure S4A and S4B). Conversely, stable overexpression of the +E9 Numb isoform under a tetracycline-inducible promotor in MDCK cells, increased the spreading area of cells on soft matrixes (Figures S4E and 6D). Together, these findings suggest that the splicing variant that contains exon 9 plays a crucial role in cell spreading on stiff matrix.

Next, we tested whether Numb isoform switching plays a role also in mesenchymal stem cell differentiation. We observed reduced osteoblastic differentiation of mesenchymal stem cells transfected with the Numb antisense oligonucleotides AON 1 or AON 4–5 compared to control transfected cells (Figure 6E). Next, we tested the role of Numb isoform switching for cell proliferation and demonstrate reduced cell proliferation after transfection of MCF10A cells with Numb antisense oligonucleotides or siRNA (Table S2), which specifically reduce the +E9 Numb isoform expression (Figures 6F, S4C, and S4D). Finally, we tested whether overexpression of Numb isoforms affects proliferation on soft ECM. We observed that while the overexpression of the ΔE9 isoform did not have any effect on the proliferation on soft ECM, the overexpression of +E9 isoform allowed proliferation on soft ECM, even to levels that were comparable to the proliferation levels observed on stiff ECM (Figure 6G). The overexpression levels of both isoforms were comparable suggesting that the observed proliferation differences are not due to differences in protein expression levels (Figure 6H). Importantly, we found similar results using MDCK cells that overexpress the +E9 isoform under a tetracycline inducible promoter but not with MDCK cells overexpressing the ΔE9 isoform (Figures S4E and S4F). Therefore, overexpression of the +E9 Numb splicing variant is sufficient to override the inhibition of proliferation in cells on soft ECM.

Our data suggest that PTBP1 facilitates stiffness-induced proliferation by promoting the expression of the +E9 isoform of Numb. To test this more directly, we used Numb isoform-specific expression constructs to perform rescue experiments in PTBP1 knockdown cells. For this, we transfected MCF10A cells with PTBP1-targeting siRNA followed by a transfection of constructs that express the Numb isoform with exon 9 (+E9) or the isoform without exon 9 (ΔE9). We observed that expression of the Numb isoform with but not the isoform without exon 9 rescued the proliferation rates of PTBP1-depleted MCF10A cells on stiff ECM to almost normal proliferation rates (Figure 6J). The PTBP1 knockdown and the overexpression of the Numb isoforms were further confirmed. Importantly, both Numb isoforms were overexpressed to comparable levels (Figure 6I). These findings are in line with the hypothesis that increased cell proliferation on a stiff matrix depends on PTBP1 promoting Numb exon 9 inclusion.

### Numb +E9 isoform regulates integrin β1 recycling in MCF10A cells

To explore the mechanism by which Numb splicing influences the mechanoresponse of cells, we investigated the established role of Numb in membrane trafficking. As shown earlier, PTBP1 regulates the ratio of Numb isoforms and controls integrin β1 surface expression. To determine whether changes in the Numb isoform ratio directly affect integrin β1 surface levels, we transfected MCF10A cells with Numb antisense oligonucleotides and conducted a cell surface biotinylation assay. This revealed a significant reduction in integrin β1 surface expression compared to control-transfected cells (Figure S5A). Specificity of surface biotinylation is confirmed by the absence of the cytosolic proteins Numb and β-tubulin from the streptavidin pulldown.

Prior research has shown that Numb can regulate receptor recycling in an isoform-specific manner, prompting us to examine its role in integrin β1 recycling.^37^ We transfected MCF10A cells with either control or Numb antisense oligonucleotides and conducted recycling assays as outlined in the STAR Methods section. Cells transfected with Numb antisense oligonucleotides exhibited delayed integrin β1 recycling compared to controls. In contrast, no significant changes were observed in transferrin receptor recycling (Figure S5B). The delay in recycling was further supported by increased co-localization of integrin β1 with the early endosome marker EAA1 following transfection with Numb antisense oligonucleotides (Figure S5C). Importantly, we detected no change in integrin β1 co-localization with the lysosomal marker Lamp1, in line with receptor recycling usually occurs from endosomes rather than lysosomes (Figure S5D).

In summary, our findings suggest that the ratio of Numb isoforms regulates integrin β1 surface expression at least in part by controlling its recycling. This mechanism is in line with the reduced integrin β1 surface levels observed on soft matrices, which, in turn, have significant and diverse effects on the mechanoresponse of cells.^38^^,^^39^

## Discussion

Here, we present a novel proteomic-based screen that allows the identification of proteins involved in mechanotransduction. Our screen identified the known and well-studied mechanosensitive transcriptional regulator YAP, which validates the approach. We further found that the subcellular localization and nuclear abundance of the splicing regulator PTBP1 is mechanosensitive, which suggested that PTBP1 can play a role in mechanotransduction. In line with this finding, emerging evidence suggests that mechanical and physical cues in the extracellular environment can control alternative splicing. For example, changes in alternative splicing have been described after mechanical loading of bone or after stretching of cells.^40^^,^^41^^,^^42^ Tissue stiffness has also been shown to regulate serine/arginine rich (SR) splicing factors, which leads to stiffness-dependent splicing of the extra domain B-fibronectin isoform which likely plays a role in tumor progression.^23^ Similarly, matrix stiffening reduces the expression levels of the epithelial splicing regulatory protein Epithelial splicing regulator (ESPR1), which regulates alternative splicing of the regulator of actin dynamics MENA, and thereby tumor cell intravasation.^43^ Thus, the mechanical regulation of alternative splicing appears to be an important principle to translate mechanical cues into cellular biochemical changes, to govern mechanosensitive cell behavior. However, the investigation of alternative splicing as an integral part of mechanotransduction is in its infancy. Here, we demonstrate that the nuclear localization of the splicing regulator PTBP1 is mechanosensitive and can be controlled by a variety of extracellular mechanical cues, and that PTBP1 is essential for the mechanoresponse of cells, including cell spreading and proliferation and likely mesenchymal stem cell differentiation on a stiff matrix. However, regarding mesenchymal stem cell differentiation we cannot rule out that our data reflect a general requirement of PTBP1 for mesenchymal stem cell differentiation into osteoblasts, which could in principle be independent of its mechanical regulation.

It has recently been demonstrated that direct application of physical force to the nucleus modulates nuclear pore permeability and affects facilitated diffusion. Furthermore, forces can be transmitted to the nucleus via the actomyosin cytoskeleton and the LINC complex leading to nuclear pore deformation and changes in permeability.^4^^,^^13^ It is known that cells display increased actomyosin contractile force when cultured on a stiff matrix.^44^ We show that lowering cellular actomyosin contractility via small molecule intervention or by placing cells on a soft matrix or small area micropattern reduces the nuclear localization of PTBP1. Hence, it is likely that a mechanically controlled nuclear permeability or mechanically controlled facilitated diffusion might contribute to the regulation of the nuclear abundance of PTBP1.

To gain mechanistic insights into the role of PTBP1 in mechanotransduction we have investigated the potential mechanosensitive regulation of PTBP1 splicing targets. We found that among other splicing targets PTBP1 regulates the matrix stiffness-dependent splicing of the endocytic adapter protein Numb. This is also accompanied by a change in protein expression of the corresponding Numb isoforms. Numb plays important roles in cell fate decisions, regulation of endocytic trafficking and lysosomal degradation of membrane receptors including Notch1, E-cadherin and the anaplastic lymphoma kinase.^45^^,^^46^ The splicing of Numb is regulated during development with the isoforms that include exon 9 expressed in stem and progenitor cells and the isoform skipping exon 9 preferentially expressed in differentiated cells.^47^^,^^48^ These isoforms have been described to serve different and partially opposite cellular functions.^48^^,^^49^ Furthermore, Numb exon 9 inclusion is increased in multiple cancers including all breast cancer subtypes. This is of particular interest given that isoforms including exon 9 promote proliferation which can contribute to cancer progression.^35^^,^^50^^,^^51^^,^^52^ Similarly, mutations in the splicing factor RBM10 lead to increased exon 9 inclusion in Numb which promotes lung cancer cell growth.^49^ Although the role of Numb isoforms in cancer is complex and appears to be cell type dependent,^53^^,^^54^ this indicates that our finding can be important for cancer progression.

Here, we demonstrate that Numb splicing crucially influences matrix stiffness-induced cell spreading, mesenchymal stem cell differentiation and proliferation phenocopying the requirement of PTBP1 for these processes. Together with our finding that overexpression of the Numb+E9 isoform can rescue the PTBP1 knockdown effect on proliferation and partially restores cell spreading on a soft matrix this supports the conclusion that PTBP1 influences mechanoresponses by regulating the alternative splicing of Numb.

Furthermore, our finding that alternative splicing of Numb, modulates cell surface levels of integrin β1 at least in part by regulating integrin β1 recycling aligns with existing evidence of reduced integrin β1 cell surface levels on a soft matrix.^38^^,^^39^ Importantly, reduced surface levels of integrin β1 are a possible but likely not exclusive explanation for the observed modulation of mechanoresponses by PTBP1 and Numb. Integrin β1 surface levels are established key determinants of cell spreading,^29^^,^^30^ and reduced cell spreading negatively impacts on YAP nuclear localization and cell proliferation, phenotypes we observed after PTBP1 knockdown.^10^^,^^32^^,^^33^ Consistently, we present data that small molecule inhibition or downregulation of integrin β1 expression is sufficient to reduce cell proliferation on a stiff matrix. Finally, integrin β1 has also been identified as essential for the osteogenic differentiation of mesenchymal stem cells in response to matrix stiffness.^55^

Notably, it has previously been described that Numb can regulate the recycling and cell surface levels of the receptor tyrosine kinase ALK in an isoform specific manner with the +E9 Numb isoform promoting recycling.^37^ Furthermore, our results are in line with a recent study that showed that the Numb +E9 isoform is important for the cell surface expression of several receptors, including integrins, which affects cell spreading.^32^^,^^33^^,^^35^ However, further experiments will be required to determine whether the effect of Numb isoforms on integrin β1 recycling is due to direct interaction with integrins or due to indirect effects on the endocytic network. Interestingly, a Numb exon 9 specific knockout in breast epithelial cancer cells causes a major remodeling of the endocytic network including the regulation of members of the recycling machinery.^35^

In summary, our data support a working model in which reduced actomyosin contractility on a soft matrix decreases PTBP1 levels in the cell nucleus. This alteration shifts the isoform expression of Numb, delaying the recycling of integrin β1 and reducing its surface levels. These changes may contribute to significant alterations to the mechanoresponse of cells (Figure S5E). This model does not exclude additional mechanisms influencing integrin β1 surface levels on a soft matrix.^38^^,^^39^

Our results have implications for tissue development and for diseases like cancer and fibrosis in which the stiffness of the tissue is higher than in healthy tissue.^56^ The targeting of alternative splicing using antisense oligonucleotides is already a clinical reality, and further knowledge of mechanoregulation of alternative splicing, might allow future development of novel strategies to mitigate disease and improve human health.^57^

### Limitations of the study

Our screen was likely not exhaustive, as we did not identify several known mechanosensitive transcription factors such as MRTF-A or Twist. This may, in part, be attributed to the choice of cell type (HEK293 cells) used for the initial screen. Our findings demonstrate that PTBP1 and the alternative splicing of Numb regulate integrin β1 surface expression levels, with Numb splicing influencing integrin β1 recycling. However, further investigation is needed to elucidate the precise underlying mechanisms. Additionally, while we confirmed a role for PTBP1 in mechanoresponses using the non-transformed breast epithelial cell line MCF10A, it will be important to assess the contributions of PTBP1 and the proposed mechanism in cellular mechanoresponses within primary epithelial cells or in a tissue context.

## Resource availability

### Lead contact

Requests for further information and resources should be directed to and will be fulfilled by the lead contact, Kai S Erdmann (k.erdmann@sheffield.ac.uk).

### Materials availability

Plasmids and stable cell lines generated in this study are available from the lead contact upon request.

### Data and code availability

• Mass spectrometry proteomics data were deposited in the ProteomeXchange Consortium via the PRIDE partner repository with the dataset identifierPXD046157. RNA-seq data were deposited in National Center for Biotechnology information’s Gene Expression Omnibus (GEO). The GEO accession number is GSE279391.
• This paper does not report original code.
• Additional information required to analyze the data reported in this paper can be available to lead contact upon request.

## Acknowledgments

We are very grateful to Sanjay Kumar and Stacey Lee, University of California, Berkeley, United States, and Manuel Théry and Benoit Vianay, Institut de Recherche Saint Louis, France, for help and advice on traction force microscopy analysis. A.S. was supported by a stipend from the Ministry of Education of Libya government. M.A. was supported through the Scholars support program of the 10.13039/100020247University of Bisha, Saudi Arabia.

## Author contributions

P.-L.T. and K.S.E. conceived the study. P.-L.T., K.S.E., A.K.B.G., and M.O.C. designed the experiments. P.-L.T., W.S., A.S., S.M., M.O.C., and M.A. performed the experiments. S.M. and A.S. performed the traction force microscopy, M.O.C. performed the mass spectrometry experiments and analyzed the data; all other experiments were performed by P.-L.T. and W.S. A.K.B.G. and S.M. analyzed the traction force microscopy data. K.S.E. and P.-L.T. wrote the manuscript. All authors commented on the manuscript with special input from A.K.B.G.

## Declaration of interests

The authors declare no competing interest.

## Declaration of generative AI and AI-assisted technologies in the writing process

During the preparation of this work the authors used ChatGPT4o in order to improve the grammar and English writing. After using this tool/service, the authors reviewed and edited the content as needed and take full responsibility for the content of the publication.

## STAR★Methods

### Key resources table

**Table:** 

REAGENT or RESOURCE	SOURCE	IDENTIFIER
**Antibodies**		

Mouse monoclonal anti-YAP	Santa Cruz	#sc-101199; RRID: AB_1131430
Rabbit monoclonal anti-YAP	Cell Signaling	#14074; RRID: AB_2650491
Mouse monoclonal anti-PTBP1	Thermo Fisher Scientific	#32–4800; RRID: AB_2533082
Rabbit polyclonal anti-Pitx2	Capra Science	#PA-1020
Mouse monoclonal anti-CCT2	Santa Cruz	#sc-374152; RRID: AB_10917207
Mouse monoclonal anti-Integrin β1	Santa Cruz	#sc-374429; RRID: AB_11012020
Rabbit monoclonal anti-CD71 (Transferrin receptor 1, TfR1)	Cell Signaling	#13113; RRID: AB_2715594
Rabbit polyclonal anti-GFP	Invitrogen	#A-11122; RRID: AB_221569
Rabbit monoclonal anti-HA tag	Cell Signaling	#3724; RRID: AB_1549585
Mouse polyclonal anti-β-tubulin	Sigma-Aldrich	#T4026; RRID: AB_477577
Mouse monoclonal anti-nucleolin	Santa Cruz	#sc-8031; RRID: AB_670271
Rabbit monoclonal anti-NUMB	Cell Signaling	#2756; RRID: AB_2154298
FITC-conjugated CD29 (Integrin β1)	Thermo Fisher Scientific	#11-0299-42; RRID: AB_2043829
Rabbit monoclonal anti-EEA1	Cell Signaling	#3288; RRID: AB_2096811
Rabbit monoclonal anti-LAMP1	Cell Signaling	#9091; RRID: AB_2687579
Mouse monoclonal anti-GAPDH	Proteintech	#60004-1-Ig RRID: AB_2107436
Mouse monoclonal anti-β-catenin	BD Biosciences	#610153 RRID: AB_397554
Mouse monoclonal anti-γ-adaptin	Lab of Dr Andrew Peden	N/A
Rabbit polyclonal anti-BrdU	Abcam	#Ab152095; RRID: AB_2813902
Mouse monoclonal anti-Ki67	Cell Signaling	#9449; RRID: AB_2797703
Alexa Fluor™ 680 Streptavidin Conjugate	Thermo Fisher Scientific	#S32358
Donkey anti-rabbit IgG (H + L) Alexa Fluor™ 680	Thermo Fisher Scientific	#A-21109; RRID: AB_2535758
Goat anti-rabbit IgG (H + L) Alexa Fluor™ 488	Thermo Fisher Scientific	#A-11034; RRID: AB_2576217
Goat anti-mouse IgG (H + L) Alexa Fluor™ 488	Thermo Fisher Scientific	#A-11001; RRID: AB_2534069
Donkey anti-mouse IgG (H + L) Alexa Fluor™ 594	Thermo Fisher Scientific	#A-11005; RRID: AB_2534073
Donkey anti-rabbit IgG (H + L) Alexa Fluor™ 594	Thermo Fisher Scientific	#A-11012; RRID: AB_2534079
DyLight™800 4X PEG conjugate anti- mouse IgG (H + L)	Thermo Fisher Scientific	#SA5-35521; RRID: AB_2556774

**Chemicals, peptides, and recombinant proteins**		

Blasticidin S HCl	Gibco	#R21001
Zeocin™ Selection Reagent	Gibco	#R25001
Hygromycin B	Gibco	#10687010
Puromycin dihydrochloride	Santa Cruz	#sc-108071
Tetracycline hydrochloride	Sigma-Aldrich	#T7660
Trypsin	Sigma-Aldrich	#B4501
Human Epidermal Growth Factor	Sigma-Aldrich	#E4127
Insulin	Thermo Fisher Scientific	#12585014
Hydrocortisone	Sigma-Aldrich	#H6909
BrdU	Merck	#203806
Phalloidin	Sigma-Aldrich	#51927
Lipofectamine™ 2000	Thermo Fisher Scientific	#11668019
Biotin	Sigma-Aldrich	#B4501
EZ-link Sulfo-NHS-SS-Biotin	Thermo Fisher Scientific	#21331
ML-7, hydrochloride	Sigma-Aldrich	#475880
Latrunculin A	Santa Cruz Biotechnology	#sc-202691
Y27632-HCl	Stratech Scientific	#s1049
MG132	Sigma-Aldrich	#474787
Doxorubicin	Cell Signaling	#5927
Leupeptin	Cell Signaling	#73618
Alkaline phosphatase (ALP) staining kit	abcam	#ab242287
Hoechst 33342, trihydrochloride	Thermo Fisher Scientific	#H3570
MycoStrip	InvivoGen	#rep-mys-20

**Deposited data**		

Proteomics Data		PXD046157: https://www.ebi.ac.uk/pride/archive/projects/PXD046157
RNA-seq Data		GSE279391

**Experimental models: Cell lines**		

Flp-In™ T-REX™ HEK293	Thermo Fisher Scientific	#R78007; RRID: CVCL_U427
MCF10A	ATCC	#CRL-10317; RRID: CVCL_0598
NIH3T3	ATCC	#CRL-1658; RRID: CVCL_0594
hMSC-BM	PromoCell	C-12974
Human Mammary Epithelial Cells	Sigma-Aldrich	830-05A
HeLa	ATCC	#CCL-2; RRID: CVCL_0030
Flp-In T-REX MDCK		N/A
HEK293-tet-RhoA-TurboID	This study	N/A
MDCK/Numb+E9	This study	N/A
MDCK/NumbΔE9	This study	N/A

**Oligonucleotides**		

siPTBP1_5	QIAGEN	SI00141638
siPTBP1_18	QIAGEN	SI02649206
siNumb (+E9)	Zhan et al.^35^^,^^54^ 2022	N/A
siNumb (ΔE9)	Zhan et al.^35^^,^^54^ 2022	N/A
siYAP	Dupont et al.^10^ 2011	N/A
siIntegrin b1	Santa Cruz	sc-35674
Stealth RNAi negative control	Invitrogen	12935300
AON scramble	This study	N/A
Numb AON1	https://patents.google.com/patent/EP3768839A1/en	N/A
Numb AON 4-5	https://patents.google.com/patent/EP3768839A1/en	N/A
Primer sequences, see Tables S3 and S4		N/A

**Recombinant DNA**		

pcDNA5/FRT/TO	Thermo Fisher Scientific	V652020
pOG44	Thermo Fisher Scientific	V600520
pcDNA3-EGFP-RhoA-Q63L	Addgene	RRID: Addgene_12968
3xHA-TurboID-NLS_pCDNA3	Addgene	RRID: Addgene_107171
pcDNA3.1 (+)-myc-PuroR	Lab of Dr Kai Erdmann	N/A
pcDNA3.1-Puro-3xHA-TurboID-NLS	This study	N/A
NUMB+E9_pcDNA3.1(+)-C-Myc	GenScript	Clone ID: OHu18046C
NUMBDE9_pcDNA3.1(+)-C-Myc	GenScript	Clone ID: OHu16398C
pcDNA5/FRT/TO-RhoA-Q63L	This study	N/A
pcDNA5/FRT/TO-Numb+E9	This study	N/A
pcDNA5/FRT/TO-NumbΔE9	This study	N/A

**Software and algorithms**		

MaxQuant 1.6.2.6	MaxQuant	RRID:SCR_014485
Perseus 1.5.6.0	MaxQuant	RRID:SCR_015753
DAVID		RRID:SCR_001881
rMATS software		RRID:SCR_023485
RStudio	Posit	RRID:SCR_000432
Image Lab Software	Bio-Rad	RRID:SCR_014210
FlowJo software v.7.6.5	BD Bioscience	RRID:SCR_008520
Fiji	Schindelin et al.^58^ 2012	RRID:SCR_002285
ImageStudioLite	LI-COR Biosciences	N/A
GraphPad Prism 9.5.1	Prism	RRID:SCR_002798
Biorender	Biorender	RRID:SCR_018361

### Experimental model and study participant details

#### Cell lines

Flp-In T-REX HEK293 cells were obtained from Thermo Fisher Scientific. MCF10A, HeLa, and NIH3T3cells were obtained from ATCC. Commercial human bone marrow mesenchymal stem cells (hMSC-BM) (female, 64-year-old donor) were obtained from PromoCell. Commercial human mammary epithelial cells (female, 28-year-old donor) were obtained from Sigma-Aldrich, University of Sheffield ethics committee authorisation, Reference No 049149. Flp-In TREX-MDCK II was a gift from Dr Jack Kaplan, University of Illinois at Chicago. HEK293-tet-RhoA-TurboID, MDCK/Numb+E9, and MDCK/NumbΔE9 were generated for this paper. Cells were regularly inspected for correct morphology and for Mycoplasma contamination using Mycoplasma Detection Kit/MycoStrip (InvivoGen) according to the manufacturer’s instructions.

#### Cell culture

Flp-In T-REX HEK293 were cultured with DMEM-GlutaMAX media (Gibco) supplemented with 10% FBS (Gibco), 1% penicillin/streptomycin (Gibco), 100 μg/mL zeocin (Gibco) and 15 μg/mL blasticidin S HCl (Gibco). Flp-In TREX-MDCK II cells were cultured with DMEM-GlutaMax supplemented with 10% FBS, 1% penicillin/streptomycin, 150 μg/mL zeocin and 6 μg/mL blasticidin S/HCl. MCF10A were cultured with DMEM/F12 media (Gibco) supplemented with 5% Horse Serum (Gibco), 20 ng/mL EGF (Sigma-Aldrich), 500 ng/mL hydrocortisone (Sigma-Aldrich), 10 μg/mL insulin (Thermo Fisher Scientific)) and 1% penicillin/streptomycin (Gibco). Human Mesenchymal stem cells from bone marrow (hMSC-BM) were cultured with Mesenchymal Stem Cell Growth Medium 2 (PromoCell). NIH3T3cells (ATCC) were cultured with DMEM-GlutaMax supplemented with 10% FBS and 1% penicillin/streptomycin. Human mammary epithelial cells were cultured with Human Mammary Epithelial Cell Growth Medium (Sigma-Aldrich). HeLa were cultured with DMEM-GlutaMax supplemented with 10% FBS and 1% penicillin/streptomycin. Cells were grown at 37°C in a humidified atmosphere containing 5% CO2, and cells of early passages were used for the experiments.

### Method details

#### Generation of vector constructs

pcDNA3-EGFP-RhoA-Q63L was a gift from Gary Bokoch (Addgene plasmid # 12968) and 3xHA-TurboID-NLS_pCDNA3 was a gift from Alice Ting (Addgene plasmid # 107171). Numb+E9_pcDNA3.1(+)-C-Myc and NumbΔE9_pcDNA3.1(+)-C-Myc isoforms were purchased from GenScript. pcDNA5/FRT/TO and pOG44 vectors were from Thermo Fisher Scientific. pcDNA3.1 (+)-myc-PuroR was generated in Dr Kai Erdmann Lab (University of Sheffield, UK).

To generate Tet-inducible constitutively active RhoA (CA-RhoA) expression vector, the full-length cDNA encoding EGFP-RhoA-Q63L was amplified from pcDNA3-EGFP-RhoA-Q63L by PCR with primers Q63L-RhoA-F (5′-CTTAAGCTTGCCATGGTGAGCAAGGGC-3′) and Q63L-RhoA-R (5′-CTTGCGGCCGCTCACAAGACAAGGCAACCAGATTTTT-3′), using Phusion High-Fidelity Polymerase (Thermo Fisher Scientific) according to manufacturer’s instructions. The EGFP-RhoA-Q63L amplicon was purified and then ligated into the pcDNA5/FRT/TO vector using HindIII and NotI sites to form the plasmid pcDNA5/FRT/TO-RhoA.

To generate TurboID expression vector pcDNA3.1-Puro-3xHA-TurboID-NLS for proximity labeling of nuclear proteomes, the cDNA region encoding HA-tagged TurboID-NLS was prepared from 3xHA-TurboID-NLS_pCDNA3 using endonuclease digestion and then integrated into pcDNA3.1 (+)-myc-PuroR vector using HindIII and XbaI restriction sites.

To generate plasmids expressing Numb+E9 or NumbΔE9 specific isoforms under the control of a tetracycline inducible promoter, cDNA region encoding Numb+E9 or NumbΔE9 were amplified from either Numb+E9_pcDNA3.1(+)-C-Myc or NumbΔE9_pcDNA3.1(+)-C-Myc by PCR with the primers FRT/TO-Numb-F (5′-CCCAAGCTTGCCACCATGAACAAATTACGGCAAAGT-3′) and FRT/TO-Numb-R (5′- CGCCTCGAGTCACAGATCCTCTTCAGAGATGAGTTTCT-3′), using Phusion High-Fidelity Polymerase (Thermo Fisher Scientific) according to manufacturer’s instructions. The purified PCR fragments were ligated into pcDNA5/FRT/TO vector using HindIII and XhoI sites to create the plasmids pcDNA5/FRT/TO-Numb+E9 and pcDNA5/FRT/TO- NumbΔE9.

#### Generation of stable cell lines

To generate HEK293-tet-RhoA-TurboID stable cell lines harboring tetracycline-inducible constitutively active RhoA and TurboID, pcDNA5/FRT/TO-RhoA-Q63L and pcDNA3.1-Puro-3xHA-TurboID-NLS were successively introduced into Flp-In T-REX HEK293 cells. In brief, Flp-In T-REX HEK293 cells were seeded in 6-well plates and allowed to grow to about 70% confluency prior to co-transfection of pcDNA5/FRT/TO-RhoA-Q63L and pOG44 using Lipofectamine2000 (Thermo Fisher Scientific) according to the manufacturer’s instructions. 24 h after transfection, the medium was changed to complete medium (DMEM-GlutaMAX containing 15 μg/mL of blasticidin S HCl) for another 24 h. Transfected cells were then cultured with selection media (DMEM-GlutaMAX supplemented with 15 μg/mL of blasticidin S/HCl, and 200 μg/mL of hygromycin B) until the cell colonies (named HEK293-tet-RhoA) were formed and collected for subsequent transfection with linearised pcDNA3.1-Puro-3xHA-TurboID-NLS. After 24 h, transfected cells were split in a 1:10 ratio and cultured with selection media (DMEM-GlutaMAX supplemented with 15 μg/mL of blasticidin S/HCl, 200 μg/mL of hygromycin B, and 1.5 μg/mL of puromycin). Single colonies were picked and expanded. Only clones exhibiting Tet-inducible CA-RhoA and biotinylation activity were selected for further experiments.

To generate MDCK/Numb+E9 and MDCK/NumbΔE9 stable cell lines, pcDNA5/FRT/TO-Numb+E9 or pcDNA5/FRT/TO-NumbΔE9 was co-transfected with pOG44 into Flp-In T-REX MDCK using Lipofectamine2000 (Thermo Fisher Scientific) according to the manufacturer’s instructions. Stable clones were selected using selection media (DMEM-GlutaMAX supplemented with 6 μg/mL of blasticidin S/HCl, and 150 μg/mL of hygromycin B) until single colonies were formed and collected.

##### Traction force microscopy

HEK293-tet-RhoA-TurboID were seeded on 35 mm 12 kPa collagen coated plates containing 0.2 μm red fluorescent beads (Cell Guidance Systems, UK) and incubated for 24 h. 30 min before imaging, the media was changed to fresh media containing 0.05 μg/mL Hoechst dye. Plates were imaged using a Cell Discoverer 7 microscope (Zeiss, Germany), maintained at 37°C, 5% CO_2_. Images of cell clusters and beads were taken before and 2 h after treatment with 1 μg/mL Tetracycline. Traction force was analyzed in FIJI.^58^ Bead displacement was determined using particle image velocimetry followed by Fourier transform traction cytometry to calculate traction force.^59^^,^^60^

#### Protein lysis and western blotting

Cells were lysed with ice-cold RIPA buffer (50 mM Tris-HCl pH 7.5, 150 mM NaCl, 0.1% (w/v) SDS, 0.5% (w/v) sodium deoxycholate, 1 mM EDTA, 1% (v/v) NP-40) supplemented with protease cocktail inhibitor (RocheProtein concentration was determined using Bio-Rad DC protein assay kit according to manufacturer’s instructions. Equal amounts of total protein were loaded onto an SDS-PAGE before transfer onto a nitrocellulose membrane (Millipore). The membranes were blocked in blocking buffer (5% skim milk, 0.05% Tween 20, 1xPBS) for 1 h, followed by incubation overnight at 4°C with primary antibodies diluted in blocking buffer and then 1 h at room temperature with secondary antibodies. Primary antibodies were used as follows: YAP (1:200; Santa Cruz, sc-101199), GFP (1:2000; Invitrogen, A-11122), PTBP1 (1:500; Thermo Fisher Scientific, 32–4800), Pitx2 (1:1000; Capra Science, PA-1020), CCT2 (1:200; Santa Cruz, sc-374152), Nucleolin (1:300; Santa Cruz, sc-8031), Numb (1:1000; Cell Signaling Technology, 2756), Integrin β-1 (1:1000; Santa Cruz, sc-374429), Transferrin receptor 1 (TfR1) (1:1000; Cell Signaling Technology, 13113), β-tubulin (1:5000; Sigma-Aldrich, T4026), γ-adaptin (1:1000; gift from Dr Andrew Peden, University of Sheffield), GAPDH (1:20000; Proteintech, 60004-1-IG), β-catenin (1:2000; BD Biosciences, 610153). Donkey anti-rabbit IgG (H + L) Alexa Fluor680 (A21109; Thermo Fisher Scientific) and DyLight800 4x PEG conjugate anti-mouse IgG (H + L) (SA5-35521; Thermo Fisher Scientific) were used as secondary antibodies for infrared detection. Streptavidin Alexa Flour 680 conjugate antibody (1:4000; Thermo Fisher Scientific) was used to detect biotinylated proteins. Results were visualized and quantified using Odyssey Sa Infrared imaging system (LI-COR).

#### LC-MS/MS analysis of nuclear proteomes

HEK293-tet-RhoA-TurboID were grown at high cell density before treatment with 500 μM biotin for 20 min at 37°C to allow the biotinylation of the nuclear proteomes. Cells were placed on ice to stop labeling followed by washing with ice-cold PBS to remove excess biotin. Cells were then lysed in ice-cold RIPA buffer supplemented with protease cocktail inhibitor and PhoSTOP phosphatase inhibitor (Sigma-Aldrich). The cell lysate was centrifuged at 16600 x g for 20 min at 4°C and the supernatant was collected. Cell lysates containing 3000 μg protein were then incubated overnight with 100 μL of Pierce Streptavidin Agarose Resins (Thermo Fisher Scientific) at 4°C. The mixture of cell lysate/streptavidin agarose was transferred to a Wizard^R^ Minicolumn (Promega) and the resins were washed with buffers in the following order: 10 mL of 2% SDS, 10 mL of RIPA buffer, 10 mL of 0.5 M NaCl, 10 mL of 2M Urea/50 mM Tris-HCl pH8, and 10 mL of 50 mM ammonium bicarbonate. The resins were then transferred to another clean Eppendorf tube and washed twice with 1 mL of 50 mM ammonium bicarbonate by centrifugation at 1800 x g for 3 min at room temperature. After removal of the supernatant, the resins were incubated in 200 μL of 50 mM ammonium bicarbonate containing 10 mM TCEP for 15 min at 37°C with shaking and next, the resins were alkylated with 4 μL of 0.5 M IAA (Iodoacetamide) for 15 min in the dark at 37°C with shaking. The samples were then digested with 1 μg of trypsin overnight at 37°C with shaking. The supernatant was transferred to a clean Eppendorf tube and acidified by adding trifluoroacetic acid to a pH of 3. The acidified samples were then desalted on Pierce C18 stage tips (Thermo Fisher Scientific) and dried in a vacuum concentrator (SpeedVac, Eppendorf). The peptides were reconstituted in 40 μL of 0.5% formic acid and 18 μL of each sample was analyzed by nanoflow liquid chromatography-tandem mass spectrometry (LC-MS/MS) using an Orbitrap Elite Hybrid Mass Spectrometer (Thermo Fisher Scientific) coupled online to an UltiMate RSLCnano LC System (Dionex). The system was controlled by Xcalibur 3.0.63 (Thermo Fisher) and DCMSLink (Dionex). Peptides were desalted on-line using an Acclaim PepMap 100 C18 nano/capillary BioLC, 100A nanoViper 20 mm × 75 μm I.D. particle size 3 μm (Fisher Scientific) at a flow rate of 5 μL/min and then separated using a 125-min gradient from 5 to 35% buffer B (0.5% formic acid in 80% acetonitrile) on an EASY-Spray column, 50 cm × 50 μm ID, PepMap C18, 2 μm particles, 100 Å pore size (Fisher Scientific) at a flow rate of 0.25 μL/min. The Orbitrap Elite was operated with a cycle of one MS (in the Orbitrap) acquired at a resolution of 60,000 at m/z 400, with the top 20 most abundant multiply charged (2+ and higher) ions in a given chromatographic window subjected to MS/MS fragmentation in the linear ion trap. An FTMS target value of 1e6 and an ion trap MSn target value of 1e4 were used with the lock mass (445.120025) enabled. Maximum FTMS scan accumulation time of 100 ms and maximum ion trap MSn scan accumulation time of 50 ms were used. Dynamic exclusion was enabled with a repeat duration of 45 s with an exclusion list of 500 and an exclusion duration of 30 s.

#### Mass spectrometry data analysis

Raw data collected from mass spectrometry were analyzed with MaxQuant version 1.6.2.6. All data were searched against a human UniProt database. Search parameters contained digestion set to Trypsin/P with maximum of 2 missed cleavages, methionine oxidation and acetylation at N-terminal peptide as variable modifications, carbamidomethylation at cysteine as fixed modification, match between runs enabled with a match time window of 0.7 min and a 20-min alignment time window, label-free quantification (LFQ) enabled with a minimum ratio count of 2, minimum number of neighbors of 3 and an average number of neighbors of 6. A first search precursor tolerance of 20 ppm and a main search precursor tolerance of 4.5 ppm was used for FTMS scans and a 0.5 Da tolerance for ITMS scans. A protein false discovery rate (FDR) of 0.01 and a peptide FDR of 0.01 were used for identification level cut-offs.

Downstream data analysis of MaxQuant outputs was performed using Perseus version 1.5.6.0 with LFQ intensities as the main categories. LFQ intensities were transformed by log2(x) and grouped according to experimental conditions (+Tet and -Tet). LFQ Intensities were normalised by subtracting the medians and missing values were randomly imputed with a width of 0.3 and downshift of 3 from the standard deviation. To identify quantitatively changed proteins between +Tet and -Tet groups, two-tailed Student’s t-tests were performed with an S0 value of 2 and a permutation-based FDR of 0.05. Gene ontology (GO) analysis was conducted by Database for Annotation, Visualization and Integrated Discovery (DAVID) (https://david.ncifcrf.gov/) against H. sapiens proteome background. Statistical significance of enrichment of GO terms were determined by adjusted *p*-values <0.05.

#### siRNA and antisense oligonucleotide transfections

siRNA and antisense oligonucleotide (AON) transfection were carried out by Lipofectamine2000 (Thermo Fisher Scientific) according to the manufacturer’s instructions. siRNA and AON used in this study are listed in Table S2. For siRNA mediated gene knockdown, cells were seeded in 35mm dish for 24 h until 80% confluence prior to transfection with a 50 nM siRNA (for MCF10A) or 250 nM siRNA (for hMSC-BM). Stealth RNAi negative control (Invitrogen) was used as negative control throughout knockdown experiments. 18 h after siRNA transfection, the medium was replaced, and the cells were allowed to recover overnight before second siRNA transfection was performed. For AON mediated changes in Numb exon 9 inclusion/skipping levels, MCF10A was seeded in 35mm dish for 24 h until 80% confluence followed by transfection with 100 nM or 200 nM AON for 24 h. Medium was changed to fresh culture medium, and cells were continued to culture for 24 h. Knockdown efficiency was verified by western blotting or immunofluorescence staining for the targeted proteins. AON mediated changes in Numb exon 9 inclusion/skipping level were assessed by RT-PCR using specific primers listed in Table S4.

#### Reverse transcription (RT)-PCR

The change of alternative splicing by various ECM stiffnesses was verified by RT-PCR. In brief, MCF10A were cultured on 35 mm soft (0.2 kPa) or on stiff (25 kPa) polyacrylamide gel (Matrigen) for five days to 50% confluence. Total RNA was isolated using RNeasy Mini Kit (QIAGEN) according to manufacturer protocol. RNA concentration and purity were measured using a NanoDrop Lite Spectrophotometer (Thermo Fisher Scientific). After DNase I treatment to remove DNA contamination, 200 ng of total RNA was reverse-tanscribed into complementary DNA with SuperScript II Reverse Transcriptase (Invitrogen). Subsequently, the cDNA was mixed with GoTaq DNA polymerase (Promega) and primers listed in Table S4, and PCR was performed on a T100 Thermal Cycler (Bio-Rad). Annealing temperatures and extension time during the thermocycling was optimised for each primer pair and size of amplicons. PCR products were analyzed by 2% agarose gel stained with SYBRE Safe (Invitrogen) and visualised using Gel Doc EZ System (Bio-Rad). DNA bands were quantified using ImageJ software. The percent splicing inclusion (PSI) of each isoform was calculated as the inclusion level (%) of the cassette exon over the sum of all isoforms detected.

#### Real-time PCR

60,000 cells of MCF10A were plated on 35 mm soft (0.2 kPa) or on stiff (25 kPa) polyacrylamide gel (Matrigen) for five days before RNA isolation using RNeasy Mini Kit (QIAGEN) according to manufacturer protocol. After removing DNA contamination with DNase I, cDNA was synthesized from 200 ng of total RNA using SuperScript II Reverse Transcriptase (Invitrogen). Real-time PCR was carried out using iQ SYBR Green Supermix (Bio-rad) on the Bio-rad CFX96 Real-Time PCR Detection System with the following PCR cycling conditions: denaturation at 95°C for 3 min followed by 40 cycles of denaturing at 95°C for 10 s and annealing and extension at 60°C for 30 s. Samples in each experiment group were run in triplicates and the relative mRNA levels were normalized to housekeeping gene ACTB. Primers used in real-time PCR are listed in Table S3.

#### RNA-seq library preparation and sequencing

MCF10A was transfected with PTBP1 siRNA (PTBP1 KD) and control siRNA (NC) for 96 h before total RNA was isolated and collected using RNeasy Mini Kit (QIAGEN) according to the manufacturer protocol. The quality and concentration of RNA were measured using a Qubit RNA Assay Kit (Thermo Fisher Scientific), and RNA integrity was analyzed using a Bioanalyzer (Agilent Technologies). RNA samples were sent to Novogene Co., Ltd, where mRNA was converted into sequencing libraries. In brief, poly(A) mRNA was enriched from total RNA of each sample using poly-T oligo-attached magnetic beads, and then fragmented and converted into cDNA. cDNA was ligated to adapters and then each library was amplified by PCR before sequencing on an Illumina platform to generate paired-end reads (150 bp). Raw sequencing data were processed using in-house Novogene scripts to remove low-quality reads and adapter sequences. Clean reads were then aligned to human reference genome (GRCh38) using HISAT2 (v2.0.5) and read counts for each gene were calculated using feature Counts (v1.5.0-p3).

#### Differential alternative splicing events analysis

RNA-seq data obtained from MCF10A with PTBP1 KD were analyzed for differential alternative splicing (DAS) using the rMATS software (v4.1.0).^61^ StringTie output files were used to identify splicing events across the transcriptome. For each event, the percent splicing inclusion (PSI) was estimated. DAS was statistically assessed using a likelihood-ratio test to calculate inclusion level differences (ΔPSI) between PTBP1 KD and control (NC) groups. Splicing events with a false discovery rate (FDR) < 0.05 and ΔPSI >0.1 were considered significant. The identified DAS were classified into five types: alternative 3′ splice site (A3SS), alternative 5′ splice site (A5SS), mutually exclusive exons (MXE), retained introns (RI) and skipped exons (SE). The volcano plot was generated using RStudio to represent differential splicing events with log2 fold change (inclusion level difference) plotted against -log10 (FDR). KEGG pathway enrichment analysis was performed to identify signaling and metabolic pathways influenced by DASs.

#### Inhibitor experiments

NIH3T3cells were plated on 12-well dish for 24 h before the treatment with various inhibitors. Cells were treated with 60 μM Latrunculin A (Santa Cruz), 80 μM ML-7 (Sigma-Aldrich), 60 μM Y27632 (Stratech Scientific) for 30 min. For inhibition of the proteasome, cells were treated with 20 μM MG132 (Sigma-Aldrich) for 3 h. The immunofluorescence staining of cells was performed as described below.

#### Immunofluorescence and image analysis

Standard immunofluorescence staining was performed by plating cells on coverslips or on collagen-I coated polyacrylamide gel (Matrigen) or on fibronectin coated micropattern (CYTOOchip). After 24 h, cells were fixed with 4% paraformaldehyde in PBS for 10 min and then permeabilized with 0.1% Triton X-100 in PBS for 15 min. After incubation in blocking buffer (0.5% FBS, 0.01% Tween 20, 1xPBS) for 1 h at room temperature, cells were incubated with primary antibodies overnight, then with Alexa Fluor conjugated secondary antibodies (Thermo Fisher Scientific) for 1 h. Primary antibodies were diluted in blocking buffer at the follows: YAP (1:200; Santa Cruz), YAP (1:500; Cell Signaling Technology), HA tag (1:400; Cell Signaling Technology), PTBP1 (1:300; Thermo Fisher Scientific), Ki67 (1:500; Cell Signaling), and nuclei were counterstained with Hoechst dye (1:1000; Invitrogen). Both primary and secondary antibody incubation were followed by three washes with 1xPBST. To visualise immunofluorescence-stained cells on coverslips, cells were embedded in Prolong Gold anti-fade mounting medium (Invitrogen). The image on coverslips were acquired with a 60x oil Len on an Olympus Fluoview 1000 Confocal microscope or with a 63x oil Len on a Zeiss Airyscan microscope, while image of cells on Matrigen was measured with a 60x water immersion Len on an Olympus Fluoview 1000 Confocal microscope. The images were processed with ImageJ software. Fluorescence intensity of YAP and PTBP1 were assessed using ImageJ and the Nuclear/Cytoplasmic ratios of target proteins were calculated using the following formula:

$$\frac{Nuc}{Cyt}of\;target\;protein=\frac{\sum -\text{nuc}/A-\text{nuc}}{\sum -\text{cyto}/A-\text{cyto}}$$

Where ∑−nuc and ∑−cyto are the sum of the intensity values of the pixels in the nucleus and cytoplasm in the images, and A−nuc and A−cyto represent the nuclear and cytoplasmic areas.

#### Co-localisation assay

MCF10A cells were seeded on 35 mm dishes for 24 h followed by treatment with 100 nM antisense oligonucleotide (AON) to repress alternative splicing of Numb exon 9. After 24 h, 0.5x10^5^ cells were replated on coverslips and continued to culture for 24 h. For detection of co-localization of EEA1 and Integrin β1, cell surface Integrin β1 was labeled with FITC-conjugated CD29 (Integrin β1) (1:500, Thermo Fisher Scientific) at 4°C for 1 h. After two washes with cold PBS, cells were placed at 37°C for 30 min to allow endocytosis of FITC-conjugated Integrin β1 followed by standard immunofluorescence staining as described above. For detection of co-localization of Lamp1 and Integrin β1, cells were pretreated with 60 μM Leupeptin (Cell Signaling) at 37°C for 2 h before labeling with FITC-conjugated Integrin β1 and then standard immunofluorescence staining. Primary antibodies used in this experiment were diluted in blocking buffer at the follows: EEA1 (1:400; Cell Signaling), Lamp1 (1:400; Cell Signaling). Coverslips were imaged on a Zeiss Airyscan microscope with an 63x oil Len. Pearson’s correlation coefficient was used to measure co-colocalization using ImageJ.

#### Osteogenic differentiation assay

hMSC-BM were plated on 12-well plates or 35 mm collagen-coated polyacrylamide gels (Matrigen) in growth medium for overnight followed by antisense oligonucleotide or siRNA transfection for 24 h. Following transfection the growth medium was replaced with osteogenic differentiation medium (Sigma-Aldrich). The osteogenic differentiation medium was renewed every 2 days for a total of 10 days of differentiation. Transfection of antisense oligonucleotides or siRNA was repeated every 3 days during the differentiation period. Bone differentiation assay was carried out by alkaline phosphatase (ALP) staining kit (abcam) according to manufacturer protocol. In brief, cells were washed with PBST before fixed with provided fixation solution for 2 min, another PBST wash and then stained with ALP for 15 min on 12-well plate or 1 h on polyacrylamide gel followed by washing three times with PBST. ALP-stained images were taken using Nikon ECLIPSE Ts2 microscope. The ALP positive area was determined with ImageJ, and the values were normalized to cell numbers quantified by Hoechst dye staining.

#### BrdU cell proliferation assay

5-bromo-2-deoxyuridine (BrdU) (Merck) immunofluorescence staining was performed to assess the influence of ECM stiffness on MCF10A proliferation. In brief, MCF10A cells of 50% confluency on polyacrylamide gel (Matrigen) were incubated with 20 μM BrdU (Merck) at 37°C for 3 h. Cells were washed with PBS followed by fixation with 4% paraformaldehyde for 15 min. After washing, cells were incubated in a permeabilization buffer for 15 min followed by treatment with 2M HCl for 5 min and then phosphate/citric acid buffer for 20 min. Cells were incubated in blocking buffer (0.5%FBS, 0.01% Tween 20, 1xPBS pH 7.4) for 1 h before being treated with anti-BrdU antibody (1:1000, Abcam) overnight at RT. After washing with PBST, cells were incubated with AlexFluor-594 conjugated secondary antibody (1:500, Thermo Fisher Scientific) and Hoechst dye (1:1000, Thermo Fisher Scientific) for 1 h. Images were measured on an Olympus Fluoview 1000 confocal with a 60x oil Len. BrdU incorporation was quantified using ImageJ. Cell proliferation was assessed as the percentage of BrdU-positive/total number of cells.

#### Cell spreading assay

MDCK/Numb+E9 stable cell lines were plated at low cell density for overnight and the overexpression of Numb exon 9 was induced by treating cells with 1 μg/ml tetracycline for 4 h. The cells were then replated on collagen I coated polyacrylamide gel (Matrigen) at low cell density for overnight (20 h) to allow cell spreading. Cells were fixed and processed with standard immunofluorescent staining described above. Cell spreading area was detected by staining F-actin with phalloidin Atto-594 (1:100; Sigma-Aldrich) and nuclei were counterstained with Hoechst dye (1:1000; Invitrogen). Images were measured on a Zeiss Airyscan microscope with an 63x oil Len and cell area was measured using ImageJ.

#### Cell surface protein biotinylation assay

MCF10A were cultured in growth medium on 35 mm dish to 80% confluence 24 h prior to label surface proteins with biotin. Cells were placed on ice and washed with cold PBS, then labeled with ice-cold 0.5 mg/mL EZ-link Sulfo-NHS-Biotin (Thermo Fisher Scientific) in biotinylation buffer (154 mM NaCl, 10 mM HEPES, 3 mM KCL, 1 mM MgCl_2_, 0.1 mM CaCl_2_, 10 mM glucose, pH7.6) at 4°C for 1 h. After one wash with cold PBS, cells were incubated with 1% BSA for 10 min on ice to quench unbound biotin and then washed twice with cold PBS. Cells were lysed with RIPA buffer (50 mM Tris-HCl, pH 7.5, 150 mM NaCl, 1 mM EDTA, 0.1% SDS, 0.5% sodium deoxycholate, 1% NP-40 with protease inhibitor cocktail (Rhoche)) on ice for 20 min. Protein lysates were collected and quantified and then 500 μg of total protein was incubated with streptavidin agarose beads (Thermo Fisher Scientific) overnight at 4°C to pull down the biotinylated proteins. The streptavidin beads were washed three times with RIPA buffer followed by boiling in 2x Laemmli buffer to release biotinylated proteins. The isolated biotinylated proteins were separated by SDS-PAGE and analyzed by western blot using indicated antibodies.

#### Biotin pulldown-based recycling assay

MCF10A in 35 mm plate were treated with 60 μM Leupeptin (Cell Signaling Technology) at 37°C for 2 h before labeled with biotin as described above, then the biotin-labelled surface proteins were allowed to internalize at 37°C for 30 min. The remaining cell surface biotinylation was stripped by 60 mM MesNa (Sigma-Aldrich) in MesNa buffer (50 mM Tris-HCl, pH 8.6, 100 mM NaCl) for 30 min on ice followed by quenching with 100 mM iodoacetamide (Sigma-Aldrich) for 15 min on ice. After three washes with cold PBS, cells were placed at 37°C for indicated time points to resume normal endocytic trafficking, followed by stripping and quenching of biotin-labelled molecules at cell surface as above. The cells were lysed with RIPA buffer (50 mM Tris-HCl, pH 7.5, 150 mM NaCl, 1 mM EDTA, 0.1% SDS, 0.5% sodium deoxycholate, 1% NP-40 with protease inhibitor cocktail (Rhoche)) on ice for 20 min. Protein lysates were collected and quantified, then streptavidin pulldown and western blot were performed as above.

#### Flow cytometry analysis

MCF10A was transfected with PTBP1 siRNA for 96 h and then trypsinized and resuspended in cold staining buffer (1% BSA/PBS). Cells were labeled with FITC-conjugated CD29 (Integrin β1) (Thermo Fisher Scientific) in staining buffer at 25°C for 30 min in the dark. After two washes with cold 1%BSA/PBS, cells were collected and resuspended in staining buffer for Flow cytometry analysis on a Invetrogen Attune NxT Flow Cytometer. Flow cytometry data were analyzed using FlowJo software (BD Bioscience, v.7.6.5).

### Quantification and statistical analysis

Graphs and statistical comparison were carried out using GraphPad Prism version 9.5.1. Unpaired t-test with Welch’s correlation were used to compare two cases and ANOVA tests were performed to analyze more cases. Data were presented as mean ± s.d. Details on experimental replicates and analysis methods are given in each figure legend. Statistical significance is denoted by asterisks∗: ∗*p* < 0.05, ∗∗*p* < 0.01, ∗∗∗*p* < 0.001, and ∗∗∗∗*p* < 0.0001. Diagrams were made using Biorender and Microsoft PowerPoint.

## Supplemental information

Document S1. Figures S1–S5 and Tables S2–S4

Table S1. Mass spectrometry results of biotinylated proteins +/− tetracycline treatment (RhoA induction), related to Figure 2
