# Supplementary material for: Mechanical control of the alternative splicing factor PTBP1 regulates extracellular matrix stiffness induced proliferation and cell spreading
Source: iScience. 2025 Mar 22;28(4):112273. doi: 10.1016/j.isci.2025.112273 (PMC12002664; doi:10.1016/j.isci.2025.112273)
Supplement: Document S1. Figures S1–S5 and Tables S2–S4 [file mmc1.pdf]

iScience, Volume *28*

Supplemental information

Mechanical control of the alternative splicing factor PTBP1 regulates extracellular matrix stiffness induced proliferation and cell spreading

Pei-Li Tseng, Weiwei Sun, Ahmed Salem, Mubarak Alaklobie, Sarah C. Macfarlane, Annica K.B. Gad, Mark O. Collins, and Kai S. Erdmann

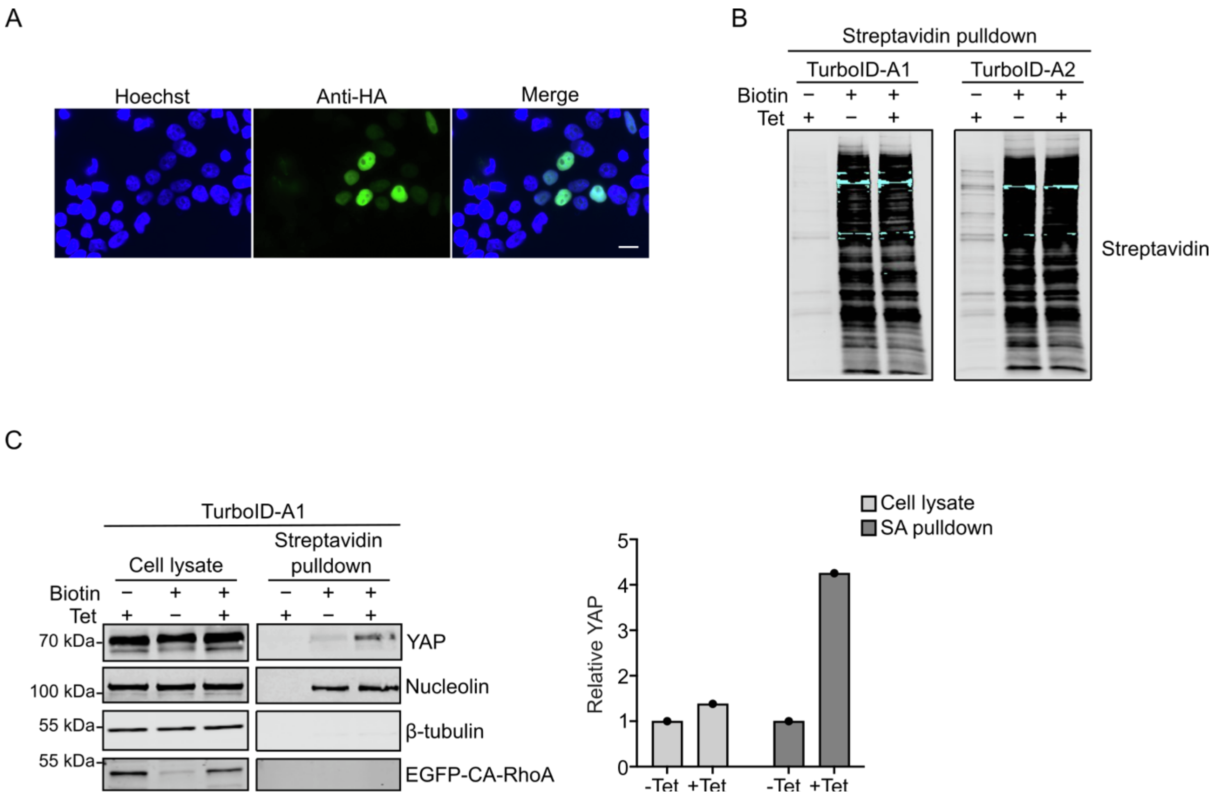

Figure S1 Validation of screen approach, related to Figure 1. A: HEK293-tet-RhoA was transfected with HA-NL-TurboID construct and nuclear localization of HA-NL-TurboID was verified by immunofluorescence. Scale bar, 15 μm. **B**: Western blot of proteins extracted from two independent HEK-tet-RhoA-TurboID clones (TurboID-A1 and TurboID-A2) showing the biotinylated nuclear proteins in cells treated with 500 μM biotin for 20 min. **C**: Western blot of indicated proteins in cell lysate and streptavidin pulldown sample from TurboID-A1 clone. Nucleolin and β-tubulin were used as nuclear and cytosolic marker respectively. Bar plot shows the quantification of nuclear YAP after tetracycline treatment. Experiment=1.

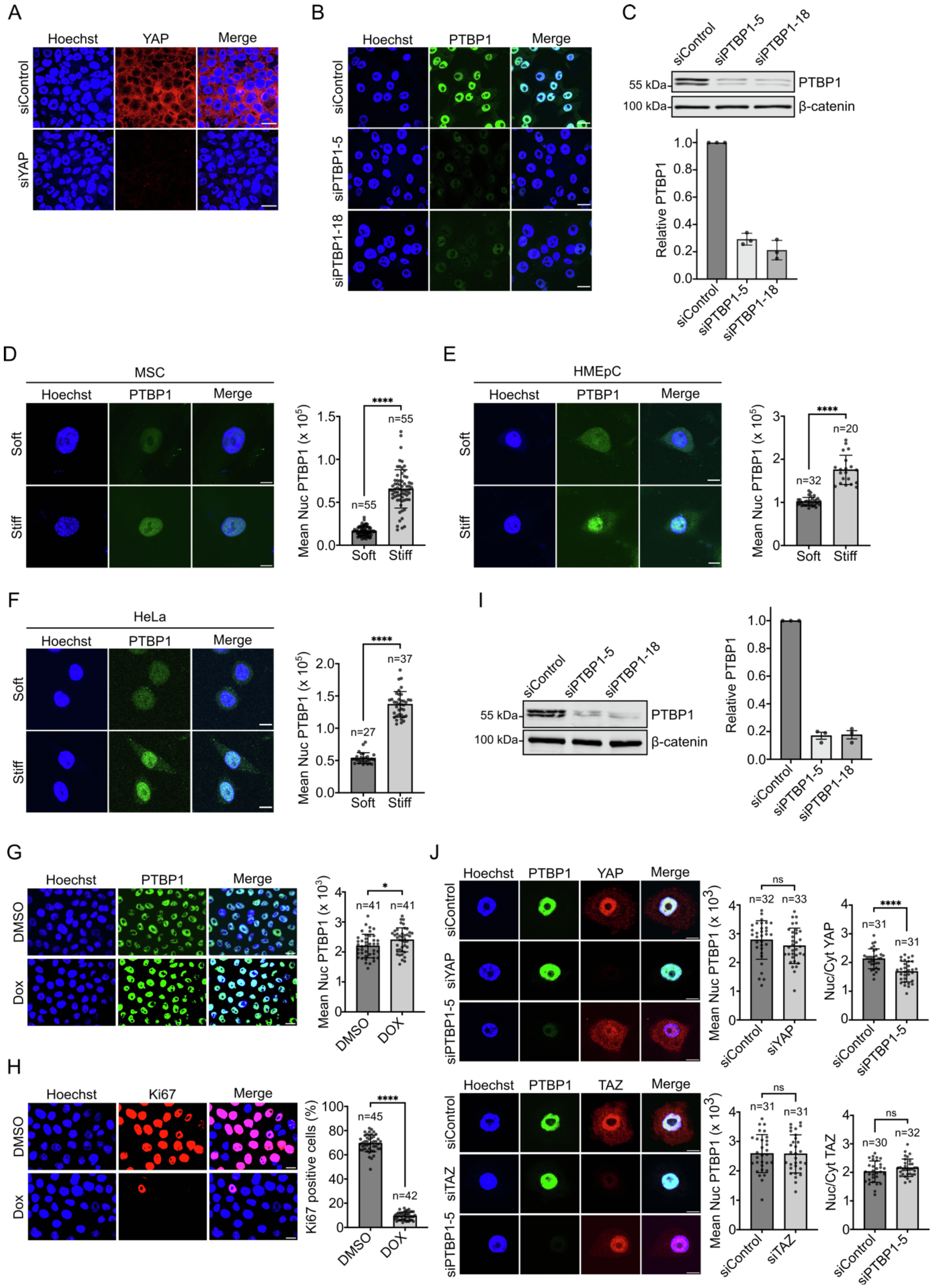

A
Hoechst
YAP
Merge
siControl
siYAP
B
Hoechst
PTBP1
Merge
siControl
siPTBP1-5
siPTBP1-18
C
siControl
siPTBP1-5
siPTBP1-18
55 kDa
PTBP1
100 kDa
β-catenin
Relative PTBP1
D
MSC
Hoechst
PTBP1
Merge
Soft
Stiff
Mean Nuc PTBP1 (x 10^5)
n=55
n=55
****
E
HMEpC
n=32
n=20
F
HeLa
n=27
n=37
I
G
DMSO
Dox
Mean Nuc PTBP1 (x 10^3)
n=41
n=41
*
DOX
H
Ki67
Ki67 positive cells (%)
n=45
n=42
J
siControl
siYAP
siPTBP1-5
Nuc/Cyt YAP
n=32
n=33
ns
n=31
n=31
TAZ
siTAZ
Nuc/Cyt TAZ
n=31
n=31
n=30
n=32

Figure S2 ECM stiffness regulates subcellular localization of PTBP1 and YAP, related to Figure 3. A: Representative immunofluorescence images of siRNA mediated knockdown of YAP in MCF10A. Bar represents 10 μm. **B**: Reduced PTBP1 expression level after siRNA-mediated knockdown in MCF10A was verified by immunofluorescence staining. Bar represents 10 μm. **C**: Western blot confirmed knockdown efficiency carried out by indicated PTBP1 siRNA. β-catenin was used as loading control. Bar diagram shows the normalized PTBP1 level after treatment of indicated siRNA. **D-F**: Mesenchymal stem cells (MSC), human mammary epithelial cells (HMEpC) (Experiment=1), HeLa cells were cultured on soft (0.2 kPa) or stiff (25 kPa) collagen coated PAA gels and nuclear PTBP1 intensity was determined by immunofluorescence. Bar represents 10 μm. **G**: MCF10A cells were treated with 0.5 μM Doxorubicin or DMSO for 72 h and nuclear intensity of PTBP1 was quantified using immunofluorescence. Bar represents 50 μm. **H**: MCF10A cells were treated as described in G and proliferation was measured by quantifying nuclear Ki67 using immunofluorescence. Bar represents 50 μm. **I**: Related to Figure 4B. siRNA mediated PTBP1 knockdown in MSC was assessed by western blot. Quantification shows the changes of relative PTBP1 level after treatment of indicated siRNA. β-catenin was used as loading control. **J**: siRNA mediated knockdown of PTBP1, YAP, and TAZ in MCF10A and nuclear intensity for PTBP1 or nuclear to cytoplasmic ratio for YAP and TAZ was measured by immunofluorescence. Bar represents 10 μm. Replicates=3 in all experiments unless stated otherwise. n= numbers of cells analysed. All data were analysed by unpaired t-test. Values are means ± s.d. *p< 0.05, ****p<0.0001.

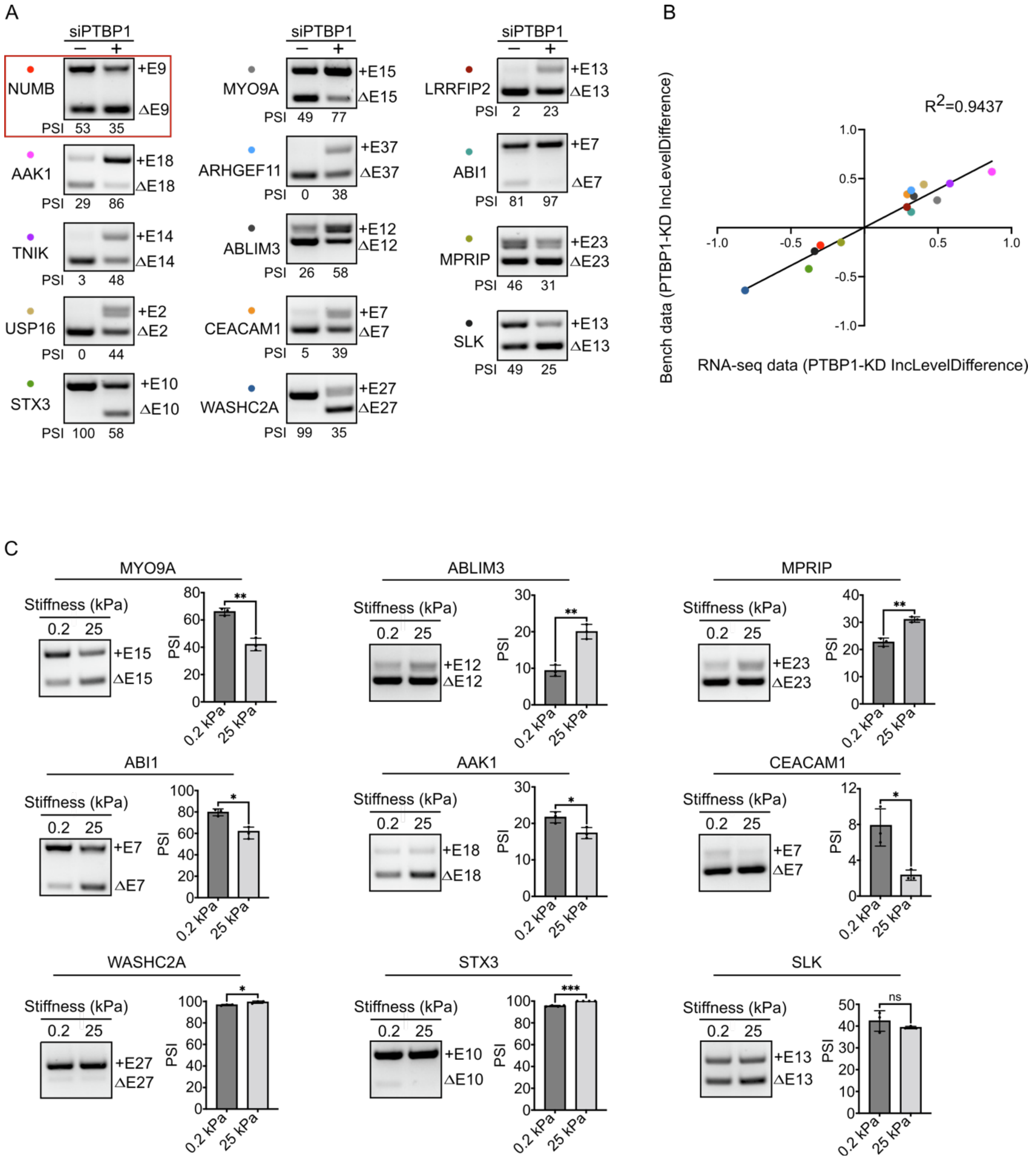

Figure S3 PTBP1 regulated alternative splicing in MCF10A cells, related to Figure 5. A: Validation of specific splicing events identified by RNA sequencing by RT-PCR. **B**: Correlation of RNA sequencing data with RT-PCR data. **C**: Selected PTBP1 regulated alternative splicing events that display stiffness-dependent regulation. MCF10 cells were cultured on soft (0.2 kPa) or stiff (25 kPa) collagen coated PAA gels for 5 days and total RNA was isolated followed by reverse transcription and analysed using PCR. Replicates=3 in all experiments. Data were analysed by unpaired t-test. Values are means ± s.d. *p< 0.05, **p< 0.01, ***p< 0.001, ns: not significant.

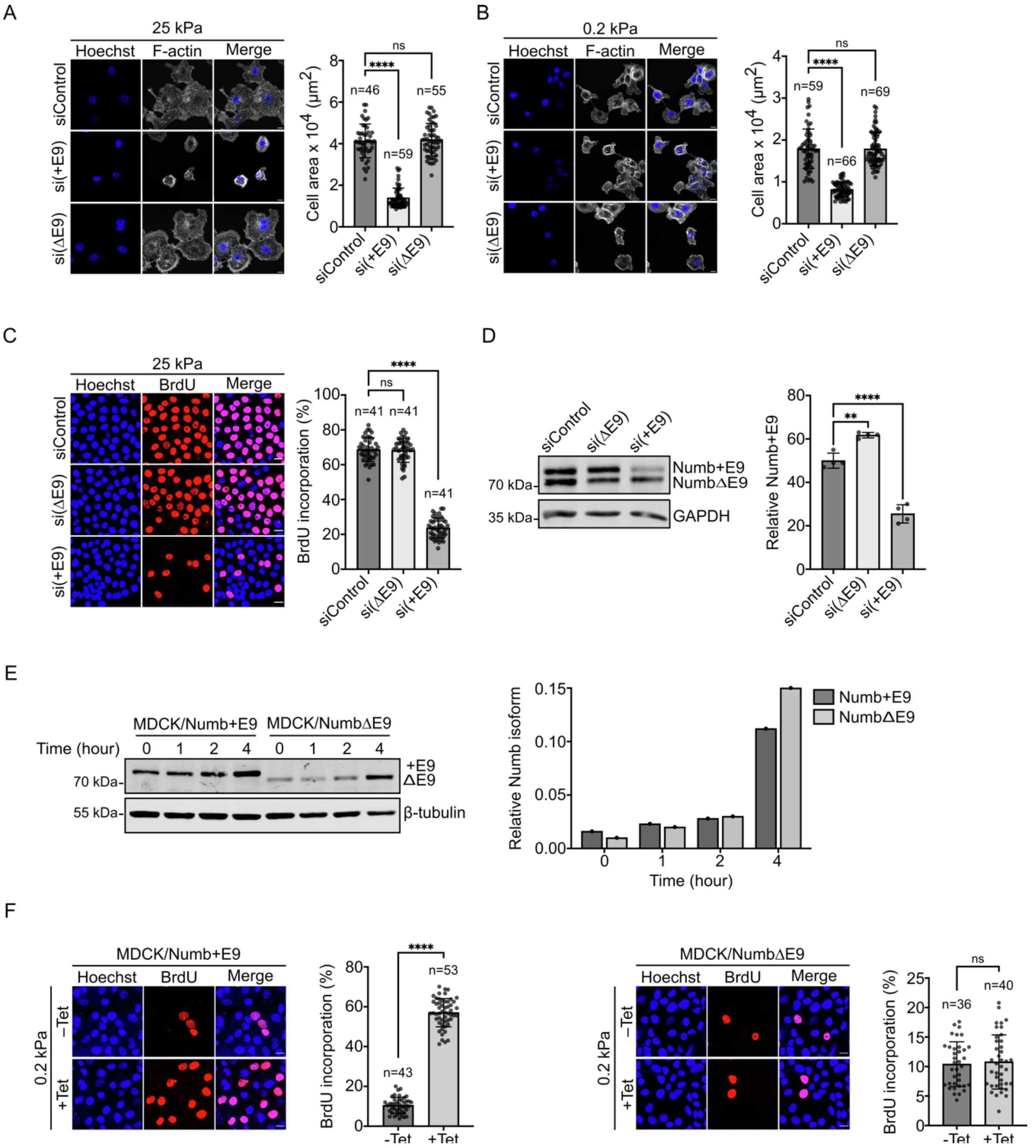

Figure S4 Numb+E9 isoform regulated ECM stiffness dependent cell spreading and proliferation, related to Figure 6. A, B: MCF10A cells were transfected with the indicated siRNAs depleting Numb+E9 isoform (si(+E9)) or NumbΔE9 isoform (si(ΔE9)) and plated on stiff (A) or soft (B) collagen coated PAA gels. Cells were stained for F-actin and nuclei and the spread area was quantified. Bar represents 10 μm. **C**: MCF10A cells were transfected with the indicated siRNAs and plated on stiff collagen coated PAA gels. Proliferation was quantified using a BrdU-assay. Bar represents 20 μm. **D**: Validation of isoform specific knockdown. MCF10A cells were transfected with the indicated siRNAs and total lysates were analysed by western blot. Quantification of Numb+E9 isoform level is defined as percentage of Numb+E9 of total Numb expression. Experiments=4. **E**: Left: MDCK cells stably expressing Numb +E9 or Numb ΔE9 isoform under a tetracycline inducible promoter were treated with tetracycline for the indicated time. Total lysate was collected and probed for Numb isoform expression by western blot. β-tubulin was used as loading control. Bar diagram shows quantification of the western blot data. Experiment=1. **F**: MDCK cells stably expressing Numb +E9 (left) or Numb ΔE9 (right) isoform under a tetracycline inducible promoter were treated with tetracycline for 4 h and cultured on soft

collagen coated PAA gels. Cell proliferation was measured by a BrdU-assay. Bar represents 20 μm. Replicates=3 in all experiments unless stated otherwise. n= numbers of cells analysed or in the case of BrdU assays refers to the number of analysed microscopic fields. Data analysis of A-D was performed by ordinary one-way ANOVA, while data of F was analysed using unpaired t-test. Values are means ± s.d. ***p*< 0.01, ****p*< 0.001, *****p*<0.0001, ns: not significant.

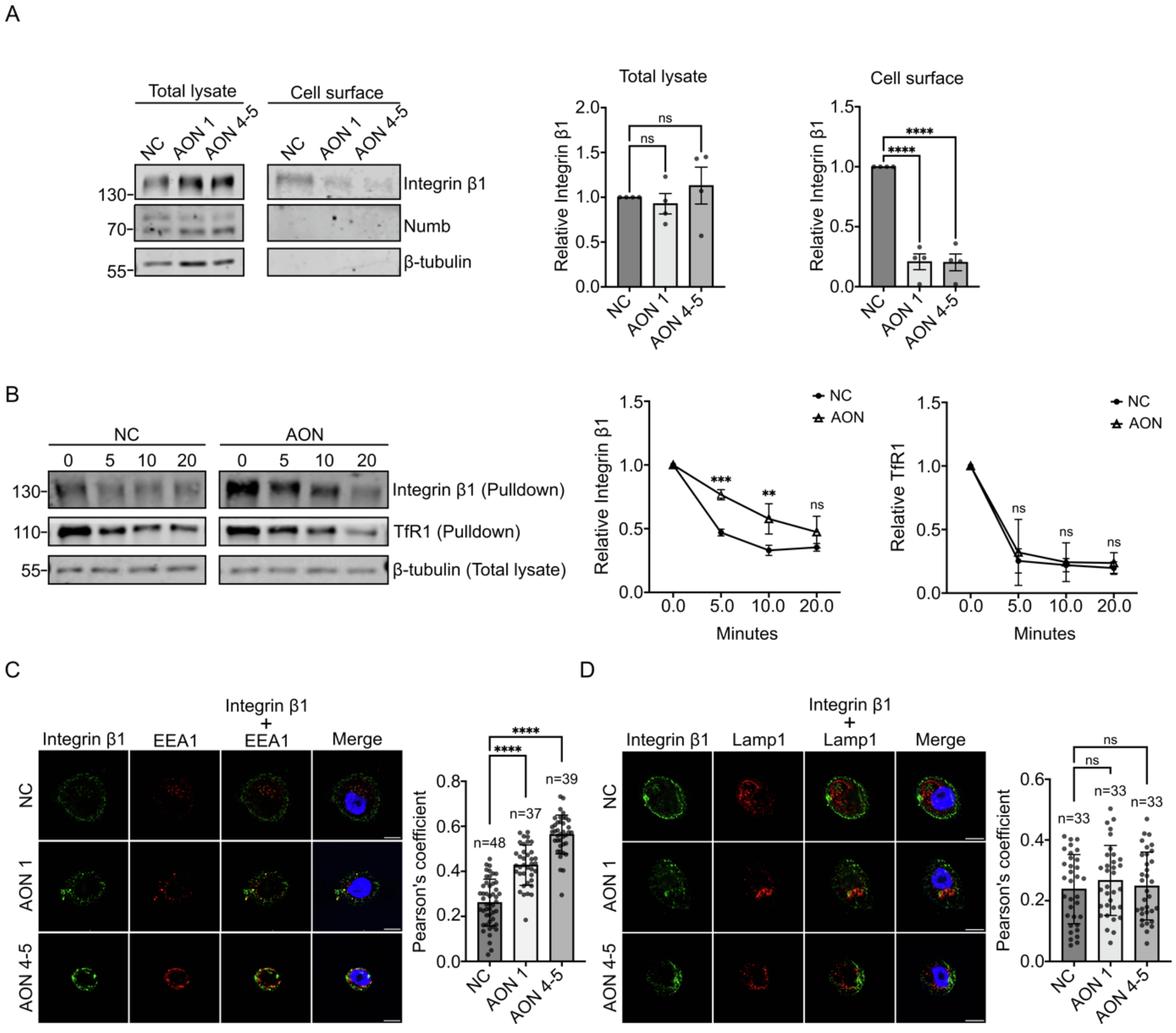

Figure S5 Numb+E9 Numb isoform regulates integrin β1 surface expression. A: MCF10A cells were transfected with the indicated antisense oligonucleotides and AON scramble (NC) followed by a cell surface biotinylation assays. Total lysates and streptavidin pulldown were analysed by SDS-PAGE followed by western blot with the indicated antibodies. Bar diagram shows quantification of total and cell surface levels of integrin β1. Data was analysed by one-way ANOVA. Replicates=4. **B**: Recycling assay of cells transfected with the indicated antisense oligonucleotides. Data was analysed by two-way ANOVA. **C, D**: MCF10A cells were transfected with the indicated antisense oligonucleotides and co-localisation of integrin β1 with the early endosomal marker EEA1 (C) or the lysosomal marker Lamp1 (D) after 30 min of endocytosis was quantified by immunofluorescence. Bar represents 10 μm. Data was analysed by one-way ANOVA. Replicates=3 in all experiments unless stated otherwise. n= numbers of cells analysed. Values are means ± s.d. *p< 0.05, **p< 0.01, ***p< 0.001, ****p<0.0001, ns: not significant.

Table S2 siRNA and RNA Antisense oligonucleotides (AON), related to STAR Methods.

Primer ID	Sequence (5'->3')	reference
**siPTBP1-5**	CGCACAUUCCGUUGCCUUATT	QIAGEN, SI00141638
**siPTBP1-18**	GCGUGAAGAUCCUGUUCAATT	QIAGEN, SI02649206
**siYAP**	GACAUCUUCUGGUCAGAGA dTdT	[S1]
**siTAZ**	ACGUUGACUUAGGAACUUU dTdT	[S1]
**siNumb (+E9)**	CACUGACUCAGCCUUCCAU dTdT	[S2]
**siNumb (ΔE9)**	ACCUUCCAAGGGACCGAGU dTdT	[S2]
**siIntegrin β1**		Santa Cruz, sc-35674
***AON Scramble (NC)**	AGUGUGUUCGGUAACUGGAUC	In this study
***Numb AON 1**	AGGCUGAGUCAGUGCCAUUAG	https://patents.google.com/patent/EP3768839A1/en
***Numb AON 4-5**	AGGGUUGGUUUCACGCACAGG	https://patents.google.com/patent/EP3768839A1/en

∗AONs used in this study contained phosphorothioate on the backbone and 2'methoxyethyl modification on the 2'sugar moiety.

Table S3 Real-time PCR Primers, related to STAR Methods.

Primer ID	Sequence	Reference
**PTBP1**	GCTGCAGAAGCCGAGTT<br>GTCTGCTCTGTGTGCCAT	Integrated DNA Technologies, Hs.PT.58.22709599
**ACTB**	CCTTGCACATGCCGGAG<br>ACAGAGCCTCGCCTTTG	Integrated DNA Technologies, Hs.PT.39a.22214847

Table S4 RT-PCR Primers, related to STAR Methods.

Primer ID	FWD sequence (5'->3')	REV sequence (5'->3')	reference
**NUMB-E9**	AGGACCCCTTCTCATCTGCT	GCACCAGAAGATTGACCCCA	[3]
**AAK1-E18**	AAGCGTGTCTGATCCTTTCAT	TGCGATTCGAGGTCACAGAT	In this study
**TNIK-E14**	ACTATGAGGAGCAGATGCGC	GCTGATGCTGAAGGGAAACTAAG	In this study
**USP16-E2**	AATTCGTCACCAGGAGGAAGA	TCATCGATTGGAACAGTTTTTCCC	In this study
**STX3-E10**	GGCACGAGATGAAACGAAAAA	GTGGTTGCAAGGAAACAAAGG	In this study
**MYO9A-E15**	TGTTTTCCGATGGGCAATTCT	TCATGCCCTGGAGATCAGAAA	In this study
**ARHGEF11-E37**	TGGACAGGAATCTGGCTGAAGAT	TCTGAGTGGTCGGTGCTTGA	In this study
**ABLIM3-E12**	ATCTACGAGAACCTGGACCTC	ACTCAGACTCCGAAGCATAGTAG	In this study
**CEACAM1-E7**	ATTGTGATTGGAGTAGTGGCC	TGAAGTTGGTTGTGTGGGTT	In this study
**WASHC2A-E27**	CATCCTGAATCCATTCAAGGTAGT	GCTCCTTCTGTGTTCAGATGAA	In this study
**LRRFIP2-E13**	TGTGTAGTGTGCTGCAGCATAA	CTCCAAGACCTGAGCAGCTT	In this study

**ABI1-E7**	AAGTAGTGGAGGAAGTGGAAGT	AGCCTGTGAGAGGTATCTGT	In this study
**MPRIP-E23**	AAAGCAACCCTGACTTCTTGA	CTCAACTTGGATGGGACACA	In this study
**SLK-E13**	GTTGTCCAAATTTCAGAATATGCTG	CTTGTTATTCAGGCACTCTCTCT	In this study

Supplemental references

S1. Dupont, S., et al., *Role of YAP/TAZ in mechanotransduction.* Nature, 2011. **474**(7350): p. 179-83.

S2. Zhan, Z., et al., *Exclusion of NUMB Exon12 Controls Cancer Cell Migration through Regulation of Notch1-SMAD3 Crosstalk.* Int J Mol Sci, 2022. **23**(8).

S3. Bielli, P., et al., *The Splicing Factor PTBP1 Promotes Expression of Oncogenic Splice Variants and Predicts Poor Prognosis in Patients with Non-muscle-Invasive Bladder Cancer.* Clin Cancer Res, 2018. **24**(21): p. 5422-5432.
